# Progress of Advanced Nanomaterials in the Non-Enzymatic Electrochemical Sensing of Glucose and H_2_O_2_

**DOI:** 10.3390/bios10110151

**Published:** 2020-10-22

**Authors:** Dayakar Thatikayala, Deepalekshmi Ponnamma, Kishor Kumar Sadasivuni, John-John Cabibihan, Abdulaziz Khalid Al-Ali, Rayaz A. Malik, Booki Min

**Affiliations:** 1 Department of Environment Science and Engineering, Kyung Hee University, Yongin 446-701, Korea; thati.daya52@gmail.com; 2 Center for Advanced Materials, Qatar University, P.O. Box 2713, Doha, Qatar; deepalekshmi@qu.edu.qa; 3 Department of Mechanical and Industrial Engineering, Qatar University, P.O. Box 2713, Doha, Qatar; john.cabibihan@qu.edu.qa; 4 Department of Computer Engineering, Qatar University, P.O. Box 2713, Doha, Qatar; a.alali@qu.edu.qa; 5 Weill Cornell Medicine-Qatar, Qatar Foundation-Education City, P.O. Box 24144, Doha, Qatar; ram2045@qatar-med.cornell.edu

**Keywords:** advanced nanomaterials, dual in-line sensing, bi-functional properties, non-enzymatic, electrochemical sensing, glucose and H_2_O_2_

## Abstract

Non-enzymatic sensing has been in the research limelight, and most sensors based on nanomaterials are designed to detect single analytes. The simultaneous detection of analytes that together exist in biological organisms necessitates the development of effective and efficient non-enzymatic electrodes in sensing. In this regard, the development of sensing elements for detecting glucose and hydrogen peroxide (H_2_O_2_) is significant. Non-enzymatic sensing is more economical and has a longer lifetime than enzymatic electrochemical sensing, but it has several drawbacks, such as high working potential, slow electrode kinetics, poisoning from intermediate species and weak sensing parameters. We comprehensively review the recent developments in non-enzymatic glucose and H_2_O_2_ (NEGH) sensing by focusing mainly on the sensing performance, electro catalytic mechanism, morphology and design of electrode materials. Various types of nanomaterials with metal/metal oxides and hybrid metallic nanocomposites are discussed. A comparison of glucose and H_2_O_2_ sensing parameters using the same electrode materials is outlined to predict the efficient sensing performance of advanced nanomaterials. Recent innovative approaches to improve the NEGH sensitivity, selectivity and stability in real-time applications are critically discussed, which have not been sufficiently addressed in the previous reviews. Finally, the challenges, future trends, and prospects associated with advanced nanomaterials for NEGH sensing are considered. We believe this article will help to understand the selection of advanced materials for dual/multi non-enzymatic sensing issues and will also be beneficial for researchers to make breakthrough progress in the area of non-enzymatic sensing of dual/multi biomolecules.

## 1. Introduction

Glucose is an essential carbohydrate involved in major catabolic pathways, including oxidative phosphorylation and glycolysis for the creation of proteins, glycogens, and lipids [1,2]. Glucose is absorbed through the intestines, and, converted by the liver into a more stable form of glycogen, regulated by the hormone insulin [3,4]. Diabetes mellitus (DM) has been termed the “invisible killer” as a consequence of both hyperglycemia and hypoglycemia [5]. A fasting blood glucose concentration less than 100 mg/dl (5.6 mmol/L) is normal, a level from 100 to 125 mg/dL (5.6 to 6.9 mmol/L) is considered prediabetes and greater than 126 mg/dL (7 mmol/L) on two separate tests allows the diagnosis of diabetes. Hypoglycemia is defined by a blood glucose concentration <70 mg/dl (3.9 mmol/L) and concentrations of both <54 mg/dL (3.0 mmol/L) and <50mg/dL (2.8 mmol/L) cause defective glucose counterregulation and impaired awareness of hypoglycemia. Hyperglycemia can result in multiple metabolic abnormalities associated with long term microvascular and macrovascular complications [6,7,8,9,10]. The global prevalence of diabetes in 2019 was estimated at 463 million people, and has been predicted to rise 10.2% by 2030 and 10.9% by 2045. The prevalence is higher in developed countries (10.4%) than in developing countries (4.0%). Furthermore, one in two people living with diabetes do not know that they have diabetes. The rising burden of diabetes in low- and middle-income countries may cause financial strain on individuals and health systems. Among all countries worldwide, the United States and China have the highest diabetes related medical expenditure. Between 2019 and 2045, the global expenditure for diabetes treatment is expected to grow from USD 760 billion to USD 845 billion. Diagnosis and management of diabetes require accurate, sensitive, reliable, rapid, and attentive monitoring of glucose in day to day life [11,12]. Generally, H_2_O_2_ is generated during enzyme/glucose reactions and so the monitoring of H_2_O_2_ is also of great importance. H_2_O_2_ is an unstable compound found in nature that plays a vital role as an intermediate in several biological reactions such as the metabolism of proteins, carbohydrates, cell signaling, and immune responses [13,14]. However, excess H_2_O_2_ can damage DNA or proteins via the generation of reactive oxygen species [15]. Hence, the monitoring of both H_2_O_2_ and glucose with a novel sensing approach in humans and the environment is of great significance. Such non-enzymatic glucose and H_2_O_2_ (NEGH) sensors have applications in biomedical devices, catalysis, and the environment. 

Several analytical approaches have been reported to quantify glucose and H_2_O_2_ levels, namely calorimetric, titrimetric analysis, spectrometry, fluorescence, chemiluminescence, and high-pressure liquid chromatography [16,17,18,19,20]. However, these methods have certain limitations, such as cumbersome fabrication processes, low reproducibility, matrix interference, high cost, and short shelf time. Hence, there is a need for the development of more efficient techniques for glucose and H_2_O_2_ quantification, and, in this context, electrochemical methods have much influence. Electrochemical techniques for glucose and H_2_O_2_ sensing have good accuracy, specificity, response time, simplicity, lower detection limits, high physical and chemical stability, enhanced electron transfer rate, practical detectability, easy to scale up, and biocompatibility [21]. The first enzyme-based glucose sensors were explored in 1960, and have served to drive work in this area for many researchers. Thereafter, first, second, and third generation enzyme-based glucose biosensors have been established. Third-generation sensors are still in their infancy, but those based on nano-mesoporous electrode surfaces show promise but with some drawbacks [22,23]. The mechanism of these sensors is based on the detection of oxygen or H_2_O_2_, the electron mediator, or the enzyme. Immobilized glucose oxidase (GOx) sensing results in the detection of gluconolactone and H_2_O_2_ [24]. Hence, the sensing of both glucose and H_2_O_2_ exists in correlation and has significance in food, pharmaceutical, clinical, and environmental studies [25,26]. However, enzymatic glucose and H_2_O_2_ sensors (EGHS) have certain limitations, including enzyme denaturation due to environmental changes (pH, humidity, and temperature), digestion by proteases, expensive preparation, time-consuming purification, high cost, thermo-chemical deformation, poor reproducibility, lack of stability, and tedious enzyme immobilization techniques [27,28]. These disadvantages of EGHS, as mentioned, can be adequately defined by nanomaterial assisted electrochemical processes through NEGH sensing.

**Figure 1:**
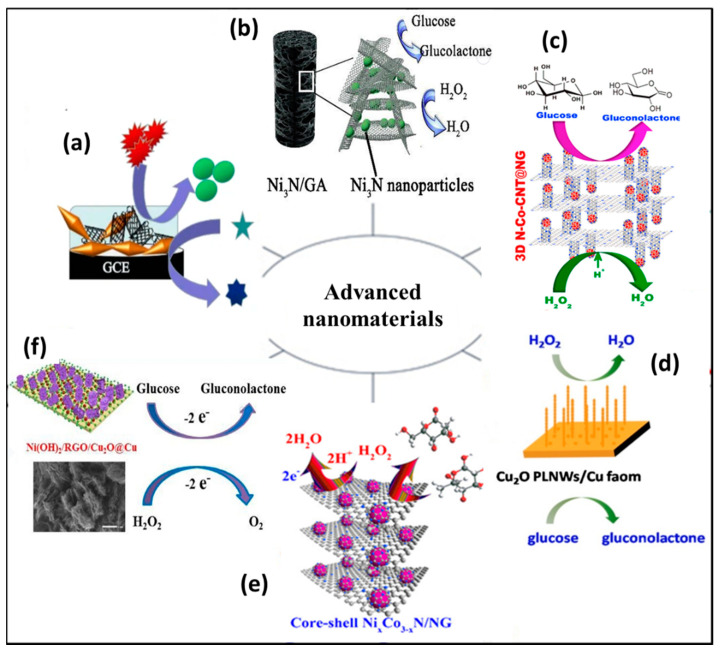
Schematic illustration of advanced nanomaterials for non-enzymatic electrochemical glucose and H_2_O_2_ sensing: (**a**) AuNBP/MWCNT/GCE nanocomposites [35]; (**b**) Ni_3_N/GA samples [36]; (**c**) 3D N-Co-CNT@NG [37]; (**d**) Cu_2_O PLNWs/Cu foam [38]; (**e**) core shell Ni_x_Co_3-x_N/NG [39]; (**f**) Ni (OH)_2_/RGO/Cu_2_O@Cu electrode [40].

The most significant challenges faced while designing NEGH sensing are the high working potential, unpredicted redox reactions, slow electro kinetics, intermediate poisoning and weak sensing parameters [29]. Therefore, recent efforts have been devoted primarily on discovering novel nanomaterials with high conductivity, efficient catalytic activity, and excellent physical and chemical strength for the construction of non-enzymatic sensors [30,31]. Nanomaterials have a large surface area, applied potential window, low charge transfer resistance, and flexibility, which makes them ideal electrode materials [32,33]. These novel nanomaterials include metal/metal oxide, carbon, and polymer nanocomposites in different nano morphologies such as crystals, rods, wires, fibers, twisters, core shell, and quantum dots (Figure 1) [34].

A wide variety of nanomaterials are fabricated; however, only a limited number of nanomaterials have been utilized for NEGH sensing due to their enhanced conductivity, surface area, electro kinetics, and the electro catalytic activity in acid, and base media. The nanoparticle concentration, synergistic effect, charge carrier type, surface charge, bandgap, mobility and density of electrons on the surface of a nanomaterial can be tuned by considering a combination of materials, and efficient preparation method, which has enabled their applications in a wide range of electrochemical devices [41,42,43]. Significant research effort was dedicated to the development of NEGH sensing with advanced nanomaterials to obtain high conductivity, suitably applied potential, and portable sensing of glucose and H_2_O_2_. Hence, this article focuses on recent advancements in the development of various nanocomposites for NEGH sensing with same electrode materials and comparatively addresses their sensing parameters in terms of wide linear range, limits of detections, response time, stability, reproducibility, sensitivity, and selectivity with critical aspects in real-time clinical, health, and environmental applications. The specific applications of different nanocomposites in real and analytical situations have been discussed and their limitations have been comprehensively addressed. Additionally, we believe that this article help to provide research directions by specifying the existing hindrances faced by advanced nanomaterial-equipped NEGH sensing and can also aid in designing novel materials. 

## 2. Metal Nanocomposites for Dual-in-Line NEGH Sensing

Most of the metal nanocomposites or hybrids benefit from their integrated properties without any alteration in structure and morphology, which can overcome limitations of the traditional noble and non-noble metals [44,45]. Technological advances in metal nanocomposite-based electrodes in several fields have stimulated their exploration in the field of NEGH sensing [46]. The ability of multiple oxidation states, stress-free oxidation of redox reactions, fast formation of intermediate compounds, and easy activation of reaction centers of metal nanocomposites is further utilized in NEGH sensing [47]. Several limitations, such as poor electrochemical activity in alkaline solutions, low diffusion of analytes towards the electrode, the solubility of the electrode, and the aggregation of metal nanoparticles during the electro catalytic process, have been efficiently addressed by the formation of nanocomposites with graphene/carbon nanotubes (both single walled and multi walled)/quantum dots/polymers. This section covers the most widely used metals and their nanocomposites for efficient glucose and H_2_O_2_ sensing. 

### 2.1. Gold and Silver Metal Nanocomposites 

Gold (Au) and silver (Ag) have shown excellent glucose non-enzymatic sensing performance because of their excellent conductivity and electro catalytic activity. These nanoparticles are notable for their antimicrobial activity and in enhancing the durability of sensors and thus are specifically significant in fabricating sensors with a longer lifetime, whereas, for H_2_O_2_, the gold-based electrode is inactive except at a very negative potential to form an O-O bond on the surface of the electrode as platinum during electrochemical sensing. Recent studies have shown O-O bond formation on the Au (100) plane surface, reflecting the different crystal facets of Au having different peroxide-like activities. In order to avoid the agglomeration issues of gold nanoparticles, carbon/polymers were used as supporting materials for electrochemical analysis. For example, Mei et al. (2019) synthesized gold nanohybrids by seed-mediated growth on Multiwalled carbon nanotubes (MWCNTs) to develop Gold Nanobipyramids (AuNBP) on MWCNTs as shown in Figure 1a. The AuNBP/MWCNT electrode showed better electrocatalytic activity than the bulk Au, Au Nanoparticle (NP), AuNBP, and MWCNT electrodes because of the more incipient gold oxide provided by AuNBPs. Electrochemical reactions in neutral pH conditions lead to glucose electro-oxidation, which is a diffusion-controlled process, whereas H_2_O_2_ reduction is a surface-controlled process. The major limitation of the AuNBP/MWCNTs hybrid is that the sensor needs to work in a strong alkaline solution to allow glucose detection. They tested the ability of the sensor to detect glucose in human serum while its ability to detect H_2_O_2_ was evaluated in antibacterial lotion (3%). Acceptable recovery with reasonable relative standard deviation (RSD) values for practical applications were reported [35]_._ Kundu et al. (2015) fabricated ordered assemblies of noble Ag NPs over Graphitic carbon nitride quantum dot (g-CNQD) sheets using the microwave assisted method. The Ag-CNx composites were assembled through an evaporation and condensation process by thermal-ultrasonic treatment. They observed superior electro catalytic activity towards H_2_O_2_ reduction/oxidation compared to 0.01 M PBS buffer than 0.05 M NaOH solution. In this work, reported H_2_O_2_ sensing at +0.7 and −0.7 V applied potential and achieved a lower detection limit of 0.6 nM (+0.7 V). Nucleation and growth of AgNPs on the voids of CNx sheets were strengthened by the Ag-N affinity and the ordered assembly of Ag particles triggered electrochemical sensing. However, the authors did not explore a wide range of molecules for selectivity and other limitations, such as low water solubility, demand further analysis of Ag NPs with the g-CNQD system [48].

### 2.2. Copper Metal Nanocomposites

The electro catalytic activity of copper metal nanocomposites is mediated by the exchange of oxidation states from Cu (II) and Cu (III) or vice versa. Economically Cu is low cost and easily available and avoids the interference compounds during sensing than the Au/Pt/Ag due to its high isoelectric point (net surface charge). Moreover, the catalytic activity of Cu-based particles are promising, making them applicable in manufacturing sensors for catalysis. Thus, major attention has been given to Cu-based electrodes for NEGH sensing in the last few years. Cu metal-based nanocomposites with different shapes and active support materials such as graphene, reduced graphene oxide, carbon nanotubes, and polymers have improved the NEGH sensing performance. The synthesis strategies of Cu-based materials improved the active surface of electrodes to form intimate contact between highly electroactive nanomaterials. During the sensing mechanism, it acts as an efficient current collector for enhancing electronic conductivity. In this regard, Babu et al. (2014) carried out work on the electropolymerization with electrodeposition technique to develop copper nanoparticles using ionic liquid on a paraffin wax-impregnated graphite electrode (PIGE). The modified electrode exhibited positive working potentials (0 V and +0.35 V) for oxidation of glucose and H_2_O_2_. A good response was achieved for glucose concentration ranging from 6.6 × 10^−6^ to 1.3 × 10^−3^ M with a detection limit of 2.2 × 10^−6^ M. The modified electrode catalyzes the electro oxidation of glucose to gluconolactone through the formation of Cu^2+^ ions. For H_2_O_2_, the electrode exhibited a rapid response in ˂4 s with a change in concentration. A linear response was achieved for 8.3 × 10^−6^ to 1.5 × 10^−3^ M with a detection limit of 2.7 × 10^−6^ M. This modified copper hybrid electrode showed the advantages of ease of preparation, excellent analytical sensing performance and carries a reduction in over potential to avoid interference. For both glucose and H_2_O_2_ detection, respective applied potentials of +0.35 V and 0 V were reported by this study, which was the least compared with concurrent studies. The practical applications for H_2_O_2_ and glucose concentrations were evaluated in solutions of stain remover and human urine samples, respectively, achieving 99.6% and 103.7% recovery rates [49]. Another research group (Mani et al., 2015) avoided the easy oxidation of Cu NPs by considering the biopolymers (pectin) as scaffold through stabilizing methods and fabricated highly stable, uniform, electroactive Cu NPs using graphene as support. The sensor displayed appreciable repeatability (five measurements), reproducibility (five different electrodes with standard deviation 2.92%) and operational stability (with 6.2% reduction in initial current when rotated in 0.1 M NaOH/2 μM H_2_O_2_ for 3000 s). The real-time applications were performed in contact lens cleaning solution and human serum for H_2_O_2_ and glucose, respectively [50]; however, reasonable data and explanations were not demonstrated. In another report, Lu et al. (2016) discussed Cu chalcogenides, i.e., sulfur-doped Cu in enhancing the sensitivity and low detection limits of glucose and H_2_O_2_. This group synthesized Cu_2_S nano rods on 3D copper foam (Cu_2_S NRs@Cu) via in situ facile electrodeposition method. The enhanced electrocatalytic activity of Cu_2_S NRs@Cu was due to its high surface-to-volume ratio and the presence of more active sites, which improved mass and electron transfer between the Cu_2_S NRs and Cu foam. In addition, it displayed ultra-high sensitivity (glucose: 11,750.8, and H_2_O_2_: 745 µA mM^−1^ cm^−2^), excellent reproducibility, selectivity, low detection limits, and also investigated real-time measurements, indicating the promising prospect for NEGH sensors in designing other biomedical applications. Stability of the sensors was explored only for two weeks, and retained the glucose and H_2_O_2_ response by 94.8% and 95.6%. These values may decrease further over time, as there is a possible degradation in the fouling resistance. This opens up the chance of more detailed analysis of the materials reproducibility and durability [51]. 

### 2.3. Nickel Metal Nanocomposites

Ni-based nanocomposite seems to be an excellent material for the fabrication of an NEGH sensor due to their attractive catalytic activity resulting from the redox/oxidation states of Ni^3+^/Ni^2+^ in alkaline media. However, the reaction mechanism is found to be different from Au-, Pt- and Ag-based electrodes. Ni-based hydroxides and oxides showed poor electrical conductivity in electro sensing. As a result, substrates with promising electron transfer ability need to be developed to sustain these active materials. Babu et al. (2013) used an ionic liquid as an electrolyte for the electropolymerization of nanomaterials on PIGE. The modified electrode was used to determine the concentrations of glucose and H_2_O_2_ along with clinically important compounds such as vitamin B6, vanillin, etc. Both cyclic voltammetry and amperometric studies were performed to study the sensing characteristics and the latter demonstrated a ˂3 s response time. Good linear range, low working potential and detection limit were achieved with effective applications in flow systems [52]. Furthermore, Wu et al. (2016) doped Ni with Sulphur for morphological change to enhance stability and reproducibility. They synthesized different phases of nickel sulfides (NiS, Ni_3_S_4_, Ni_7_S_6_, Ni_9_S_8_) by a facile hydrothermal method using thiourea and ethanolamine. Among them, they obtained 3D flower-like Ni_7_S_6_ for NEGH sensing. The cyclic voltammetry (CV) graph Ni_7_S_6_/Glassy carbon electrode (GCE) contained two redox peaks, which explained the electrocatalytic mechanism 0.1 M NaOH. The first reduction peak was attributed to the conversion of Ni_7_S_6_ to Ni_7_S_6_OH. After 20 cycles, a second reduction peak appeared corresponding to the conversion of Ni(OH)_2_ to NiOOH. The first reduction peak gradually became weaker, but the second reduction peak gradually became stronger, indicating that Ni_7_S_6_ was consumed gradually and converted to Ni(OH)_2_. The applicability of the sensor for H_2_O_2_ sensing in antibacterial lotion (3%), and for glucose in human serum samples was evaluated [53]. The values calculated for the glucose in serum was 5.55–5.64 mM, in close proximity to the glucometer data (5.60 mM), and with 4.51% to 3.28% relative standard deviation. To further enhance the electrocatalytic activity of Ni_7_S_6_, the same research group doped different concentrations of cobalt (x = 0.5, 1.0, 1.5, 2.0, 2.5, 3.0 and 3.5). Among the synthesized compounds, Ni_5.5_Co_1.5_S_6_ had shown high sensing performance having an aloe-like morphology. This sensor evaluated glucose and H_2_O_2_ in antibacterial lotions and water samples (lake/tap/pickle water) and showed a reasonable recovery rate of 99–103% for glucose, H_2_O_2_ and nitrite [54]. To avoid the drawback of sulphur poisoning during real-time application, the material was doped with nitrate, and, to enhance conductivity and stability, Yin et al. (2018) prepared novel Ni_3_N NPs on conductive Graphene aerogels (GA) via hydrothermal method cum freeze-drying and calcination under NH_3_ atmosphere. Figure 1b represents the Nickel nitride (Ni_3_N)/GA-modified electrode for NEGH sensing. The authors were successful in demonstrating the influence of structural characteristics on the sensing performance of the Ni_3_N/GA sample. The three-dimensional aerogel provides multifunctional electronic or ionic pathways through their interconnected macroporous framework and allows for the easy transportation of electrons and ions. In addition, the aerogels prevent Ni_3_N aggregation and thus increases the active sites for electrocatalysis. In short, the results demonstrated that the prepared sensor with an efficient conductive nature is applicable for perfect charge transfer and avoided the agglomeration problems, which is a huge benefit for non-enzymatic electrocatalytic application [36].

### 2.4. Cobalt Metal Nanocomposites

**Figure 2:**
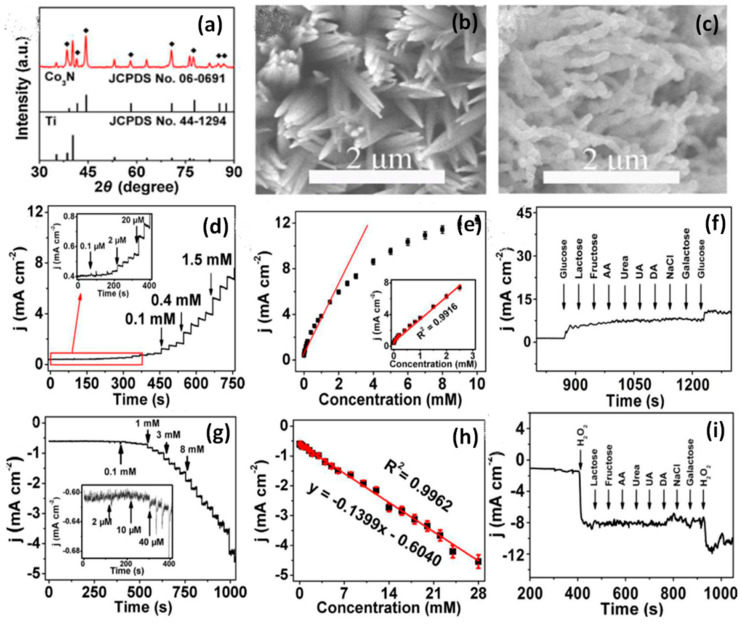
Co_3_N NW/TM: (**a**) XRD pattern; (**b** and **c**) SEM images of Co (Co(OH)_2_/TM; (**d**) ampherometric i-t response of Co_3_N NW/TM at 0.55 V (vs Hg/HgO with successive addition of glucose with varying concentration from 20 μM to 5.5 mM); (**e**) corresponding calibration curve of Co_3_N NW/TM for the detection of glucose; (**f**) interference studies in the presence of glucose; (**g**) ampherometric i-t response of Co_3_N NW/TM at 0.55 V (vs Hg/HgO with successive addition of H_2_O_2_ with varying concentration from 20 μM to 5.5 mM); (**h**) corresponding calibration curve of Co_3_N NW/TM for detection H_2_O_2_; (**i**) interference studies in the presence of H_2_O_2_ [56].

The Co-based catalysts for NEGH sensing was explored and the effect of different morphological structures on electroanalytical properties, and ideal support material for active catalyst loading was studied. As of now, various Co-based oxides, phosphides, sulfides, and complex structures with carbon/polymers have been explored for non-enzymatic monitoring of glucose and H_2_O_2_. Electrochemical sensors based on transition metal sulfides offers a more active and cheaper catalyst for sensing both glucose and H_2_O_2_. Among various metal sulfides, Cobalt sulphide (CoS) has attracted intense research interest due to their outstanding physical and chemical property with excellent catalytic properties and have been excellent in glucose and H_2_O_2_ detection. In this direction, Wu et al. (2017) studied the ability of different phases of cobalt sulfides to sense glucose and H_2_O_2_. One-pot hydrothermally synthesized CoS had a tremella-like nanostructure, and the sensor based on this material exhibited simple operation, good selectivity, stability, and reproducibility [55]. The good electrochemical response towards glucose and H_2_O_2_ was due to the absorption of intermediate species with higher glucose concentration on the electrode surface. The CoS sensor was compared with different H_2_O_2_ and glucose sensors. The CoS sensor showed 17 times wider range of detectability than NP-PtCo and 24 times higher value than nano CoPc-Gr. The detection limit (1.5 µM) was also comparable with the other sensors. Recovery of the sensors from the detection of human serum sample was also appreciable with a relative standard deviation of 2.82%. Furthermore, a novel, scalable, and one-pot method was illustrated by Balamurgan et al. (2017) to prepare a 3D N_2_ doped Co-CNT over the graphene sheet (3D N-Co-CNT@NG). This novel biosensor contains a porous architecture with a high conductive nature for efficient charge transfer at low oxidation and reduction potentials. The pictorial representation of 3D N-Co-CNT@NG for NEGH sensing is shown in Figure 1c. The synergetic interaction between metallic cobalt and nitrogen-doped CNT provided an outstanding electrocatalytic activity, and the fabricated sensor showed promising application in human serum samples and had great potential in health and environment applications [37]. To improve the performance of Co-based NEGH sensing, Fengyu et al. (2018) developed a high electroactive electrode by utilizing a cobalt nitrate nanowire array on Ti mesh. They produced Co_3_N nanowires (NW) array by hydrothermal with NH_3_ gas heat treatment method using Co (NO_3_)_2_·6H_2_O, NH_4_F, and urea as source materials and found that the array was embedded with Co, N, and Ti ions. The X-ray diffraction (XRD) profile, Scanning electron microscope (SEM) images, corresponding CV curves, ampherometric i-t response, and interferences study results show the structural and morphological features for stable sensing performance of Co_3_N NW/Titanium mesh (TM) for glucose and H_2_O_2_ detection as shown in Figure 2a–i. The non-enzymatic Co_3_N NW/TM sensor reported a 0.1 μM to 2.5 mM detection range, a 50 nM low detection limit, a 3325.6 μA mM^−1^ cm^−2^ response sensitivity and a ˂5 s response time for glucose. For H_2_O_2_, the detection range was from 2 μM to 28 mM with a 1 μM limit. This study suggests a low cost, simple preparation method to prepare 3D doped nano array for NEGH sensing applications, practically useful in electronics and catalytic devices [56].

### 2.5. Other Metal Nanocomposites

Co, Cu, and Ni are the most studied transition metals for generating electrochemical sensors for glucose and H_2_O_2_ [56,57,58]. However, a few other metals are also reported for their role in developing sensing elements [59]. Barman et al. (2016) obtained improved electron transfer coefficient and catalytic rate constant for the prepared vanadium-based samples. In this work, they modified a gold electrode using a bis(acetylacetonate) oxo vanadium (IV) transition metal complex with 4-(pyridine-40-amido) thiol phenol (PATP) for NEGH sensing in neutral medium. This sensor showed good selectivity in the presence of AA, UA, L-dopa, L-Cysteine and Na^+^, K^+^ and Cl^-^ ions. It obtained an excellent recovery rate in human serum for glucose and for H_2_O_2_ in processed milk samples. This work provided a simple preparation process, stability, and low-cost sensor for the clinical and food industry [60]. The study also addressed the influence of scan rate, accumulation potential, time, and pH on the electrochemical sensing properties of the electrode. High scan rates shift the oxidation peak potential of glucose to more positive and reduction potential of H_2_O_2_ to more negative which confirms the kinetic limitation of the electrochemical reaction. When the accumulation time was changed from 0 to 300 s, the oxidation peak currents of both glucose and H_2_O_2_ remained the same; however, the accumulation potential variation (to more positive) decreased the glucose peak current due to oxidation. For H_2_O_2_, the change in accumulation potential to a negative value caused reduction. Both the oxidation peak potential of glucose and the reduction peak potential of H_2_O_2_ were pH dependent and they respectively shifted to more negative and more positive with increased pH values (5–10). Good reproducibility (relative standard deviation of 0.2% for glucose and 0.3% for H_2_O_2_, in 10 repeated cycles), sensitivity (120.24 µA cm^−2^ mM^−1^ for glucose and 326.66 µA cm^−2^ mM^−1^ for H_2_O_2_), stability (retained 100% response after 20 days) and selectivity were achieved, making it applicable in clinical diagnosis and the food industry. 

Sarkar et al. (2018), developed sensors using transition-metal dichalcogenide-based vanadium sulfide (VS_2_) via template free-solvothermal decomposition process and utilized vanadium for the first time to study sensing parameters. The developed sensor electrode reported a selective and sensitive non-enzymatic detection of H_2_O_2_ with a sensitivity of 41.96 μA mM^−1^, linear range of 0.5 μM to 2.5 mM with a lower detection limit of 0.224 μM. The high conductivity, abundant source, and low cost of VS_2_ NPs motivated to study NEGH sensing [61]. Tian et al. (2013) converted bulk C_3_N_4_ into ultrathin graphitic carbon nitrate (g-C_3_N_4_) using ultra sonication-assisted liquid exfoliation, which offered a low-cost synthesis method with an efficient electro catalyst for NEGH sensing. The modified g-C_3_N_4_ Nano sheet/GCE showed enhanced electro catalytic activity at a very low negative operational potential of −0.60 V towards H_2_O_2_. In the same way, amperometric responses towards glucose were obtained at 0.81 V. It is important to note that g-C_3_N_4_ nano sheets have advantages over noble metal nanomaterials in the form of low-cost fabrication and bulk preparations of samples. It also showed a detection limit of 11 and 45 µM, respectively, for the buffer and human serum media [62]. 

This section reviews the significant roles of metals such as Au, Ag, Cu, Ni, Co and V in combination with MWCNTs/graphene/reduced graphene/graphitic carbon nitrate/biopolymers in NEGH sensing. The development of simple preparation techniques and attention towards transition metal chalcogenides (TMDs) such as NiN_2_, CoN_2_, Cu_2_S, CoS and V_2_S has overcome the limitations of poor conductivity, less exposed active sites, poor measurement stability, low contacted target analytes, low electron transfer, chemical instability, wide band gap and also reduced over potential issues. In a few reports, the modification of carbon materials into reduced graphene oxide, 3D graphene aerogels, and graphitic carbon nitride with unique geometry has improved porous structure for electron/ion transfer, high conductivity, strong adhesion property to catalyst particles, high mechanism strength, thermal stability, agglomeration of nanoparticles, etc., and favored reproducibility. The development of an advanced electro deposition process, and in situ fabrication techniques for metal nanocomposites has improved long-term stability for the NEGH sensors. The detection of H_2_O_2_ requires high over potentials, which in turn causes interference issues and decreases the sensitivity. These can be overcome by considering efficient synthesis strategies, such as the aforementioned synthesis methods. The working potential of modified electrodes is a key parameter for dual sensing application, which was effectively changed by modifying the morphology of metal nanocomposites into sheets, nano wires, nano rods, and flower-like structures that enhanced the surface to volume ratio to increase mass and electron transfer issues. Overall, the metal nanocomposites performed excellent catalytic activity, and exhibited notable NEGH sensing performance. By doping the different concentration of metals in sulfides and nitrides, especially transition metals can further improve the sensitivity, detection limits and linear ranges of glucose and H_2_O_2_ analytes. The sensing performance of metal nanocomposites in alkaline and acid conditions are still not clear and need to be improved further by considering core-shell-like nanostructure morphologies. Electrochemical sensing parameters such as sensitivity, detection limits, linear ranges, working potentials, storage stability, repeatability, reproducibility and real-time applications are compared for different modified metal nanocomposite electrodes for both glucose and H_2_O_2_ sensing, in Table 1.

## 3. Metal Oxide Nanocomposite for Dual-In-Line NEGHS

Metal nanocomposites had limitation for NEGH sensing such as inferior performance under neutral or low pH conditions and easy oxidation in harsh environments because of the dependency of MOOH species on the electro oxidation/reduction of glucose and H_2_O_2_. These limitations have increased focus on the development of metal oxide nanocomposites in NEGH sensing. Metal oxide sensors have the advantages of rapid electro catalytic response with specific morphology of nanoparticles, nanotubes, nanowires, nanofibers, graphene/CNTs, among others. In this section, we discuss advanced developments of NEGH sensors based on various metal oxides. In Table 2, the information on metal oxides for NEGH sensors is reported, and a brief comparison of H_2_O_2_ and glucose with the same electrode materials is given in terms of the sensing parameters.

### 3.1. Copper Oxide (CuO) Nanocomposite

**Figure 3:**
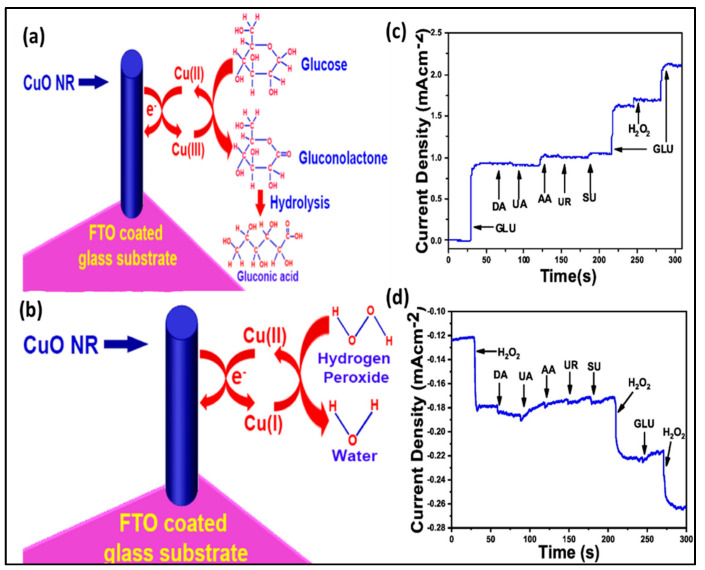
Schematic representation of CuO NRs: (**a**) glucose oxidation; (**b**) H_2_O_2_ reduction; (**c**) interference study during glucose sensing after the addition of 0.1 mM of DA, UA, AA, UR and SU and 0.5 mM of H_2_O_2_ along with 0.5 mM glucose; (**d**) interference during H_2_O_2_ sensing during the addition of 0.1 mM DA, UA, AA, UR, SU and 0.5 mM of glucose along with 0.5 mM H_2_O_2_ [66].

The natural abundance, low cost, and unique optical and electro catalytic properties of CuO mark them as one of the suitable nanomaterials for heterogeneous catalysis, magnetic storage devices, lithium-ion electrodes, gas sensors, and photovoltaic devices. Compared with the unstable Cu and Cu_2_O, CuO nanostructures are relatively stable for electro sensing analysis. NEGH detection technology can be used to design CuO nanomaterials with enhanced non-enzymatic intrinsic characteristics [63]. They are synthesized in the form of nano spheres, rods, wires, and flowers. Prathap et al. (2012) conducted a study to control the morphology of copper oxide (CuO) using different acids such as ammonia/citric/tartaric acids via the hydrothermal method and proposed a CuO formation mechanism based on the experimental results. According to the mechanism, crystal formation fully depends on nucleation and crystal growth. The addition of NH_3_ and NaOH to the reaction medium forms a Cu(NH_3_)_4_^2+^ complex followed by the precipitation of orthorhombic Cu(OH)_2_. This is in the form of a sheet-like structure connected through H-bonding, the length of which enhances with amino acid interaction. During the hydrothermal reaction, the amino acid functional group forms a co-ordinate bond with Cu^2+^ resulting in its adsorption on the crystal particle surface, preventing the re-dissolution/re-precipitation. This causes formation of flower-like morphologies compared to the dumbbell morphology during normal reaction conditions. In fact, the chemical nature of acids and hydrothermal time modified the morphology of CuO. The tyrosine amino acid synthesized CuO showed the best electro catalytic activity in this study and the results were compared with conventional CuO nanoparticles. This work provided new insight for the fabrication of CuO with different morphologies using different chemical additives and demonstrated the influence of large specific surface area and porosity in enhancing electron transfer and thus sensitivity [64]. Recently, Liu et al. (2019) developed a novel electrochemical sensor with hollow CuO/Polyalanine (PANI) nano-hybrid co-axial fibers via. electrospinning using poly(acrylic acid)(PAA) as a sacrificial template. The utilization of PANI in this work achieved excellent stability, high specific capacitance, strong adsorption, large surface area and many reactive sites. The three-dimensional porous structure of the developed sensor elements and the hollow structure of the hybrid nanofiber enhanced the surface area and the reactive sites and enabled the electrochemical sensing at ultra-low concentration levels. The developed electrode also retained its initial current response after 10 days and showed a promising application in clinical and food testing [65]. In addition, Chakraborty et al. (2019) synthesized 1D nanomaterials (CuO nanorods) over Fluorine doped Tin Oxide (FTO) substrate via the novel hydrothermal method and suggested that the 1D nanostructure electrodes are favorable to NEGH sensing due to their low fabrication cost, high electro active surface area, and excellent charge transfer property compared to other nanostructures. The pictorial representation of glucose oxidation, H_2_O_2_ reduction, and interference studies of this work are shown in Figure 3a–d. In glucose sensing, the high valence Cu^3+^ mediates the electro oxidation of glucose on the CuO surface. This happens when the glucose oxidation converts Cu^2+^ to Cu^3+^ and the formed ion acts as an electron delivery system for the glucose-gluconolactone-gluconic acid conversion. Similarly, the electro catalytic reduction of H_2_O_2_ reduces Cu^2+^ to Cu^1+^, which intermediates the H_2_O_2_ to water conversion. This group accurately performed simultaneous sensing of glucose in the presence of H_2_O_2_. The data demonstrated negligible current density changes with the addition of interfering agents compared to the current density variation with glucose/H_2_O_2_ addition. Thus, the dual sensor developed with a stability of 30 days was observed to be useful in practical applications from the point of manufacturing biodevices [66].

### 3.2. Cuprous Oxide (Cu_2_O) Nanocomposite

Cu_2_O is a well-known p-type semiconducting material with a 2.17 eV band gap and is applied in many potential applications, such as lithium ion batteries, solar cells and gas sensors. Zhang et al. (2009) provided a promising Cu_2_O microstructure for NEGH sensing application. They fabricated porous cuprous oxide (Cu_2_O) microcubes by a simple sonochemical route and its sensing results were compared with smooth surface Cu_2_O microcubes under similar experimental conditions. The porous cubes had much higher performance compared to that of the smooth Cu_2_O attributing to the porous microstructure, which provided abundant active sites for glucose and H_2_O_2_ sensing [67]. Li et al. (2011) implemented a low-temperature chemical method for the preparation of hierarchical Cu_2_O nanocrystals with the help of sodium borohydride (NaBH_4_), polyvinyl pyrrolidone (PVP) and N,N-dimethylformamide (DMF). The high charge-transport channels in hierarchical Cu_2_O nanocrystals was due to the self-assembly of nanocrystals and the presence of many grain boundaries with a compact attachment of nanocrystals. The increased electro active surface area showed a fast amperometric response and sensitivity for H_2_O_2_, which was much higher than glucose detection. The response time of less than 0.5 s was required to achieve steady current during H_2_O_2_ detection with 0.39 × 10^−7^ mol L^−1^ detection limit. However, the developed sensor showed 1.2 × 10^4^ times higher detection limit for the glucose compared with the H_2_O_2_, the reason for which was not fully addressed [68]. In another work, Gao et al. (2012) successfully prepared mesocrystalline Cu_2_O hollow nanocubes (MCHNs) via a facile reduction reaction and studied the effects of reaction parameters. To identify factors contributing to unique characteristics for the formation of MCHNs, experiments were performed by changing CuCl_2_ to CuSO_4_. Hierarchical mesoporous spheres were formed with CuSO_4_. At the same time, when LiOH was changed to NaOH, a cubic shaped product with a solid or hollow appearance was obtained, suggesting the leading role of Cl^−^ ions in the formation of distinctive MCHN structure. By varying the temperature, the final product was analyzed at low and high temperatures, and the formation of nanocubes was observed with some wide size distributions. These results confirm that the kinetics of reactions are essential for the formation of different morphologies of Cu_2_O products. Finally, they showed high resistance to interference species with excellent reproducibility and high stability [69]. In another study, Liu et al. (2013) improved the electrochemical cycling stability of Cu_2_O nanocubes by wrapping with graphene. The resulting nanocomposite showed a glucose-sensing response with a low detection limit of 3.3 µM and a linear response of 0.3 to 3.3 mM. The non-enzymatic H_2_O_2_ sensor exhibited an electrocatalytic response with a linear range of 0.3 to 7.8 mM and a low detection limit of 20.8 µM. While other studies use interferences with 1/20 to 1/10 glucose concentration to study the selectivity of the sensor, this study tested interferents with 1/2 glucose concentration in 0.1 M KOH. Lower potentials generated negligible current responses, and at 0.7 V, the responses become ˂3.5%, which is a comparatively good sign of selectivity. Moreover, the high chloride tolerance was also confirmed for Cu_2_O/GNs as it did not change the current of glucose oxidation. The very good linear response, selectivity and detection range are associated with the higher electron transfer ability and increased electro catalytic surface area [70]. In another reearch study, Cu_2_O was combined with carbon quantum dots (CQDs) to enhance the stability and sensitivity for NEGH sensing. The Cu_2_O/CQDs were synthesized by a hydrothermal with ultrasonic treatment method, and the presence of low-index (111)-octahedral planes showed good electrochemical performance and stability in the sensing of all low-indexed planes. The scan rate also affected the glucose oxidation, as increasing scan rates increased both oxidation and reduction currents. The water solubility and biocompatibility of CQD with octahedral Cu_2_O further enhanced linear response ranges and selectivity issues. In short, the CQDs/octahedral Cu_2_O/Nafion/GCE provided wider detection range, shorter detection limit and response time than the octahedral Cu_2_O/Nafion/GCE, attributed mainly to the synergistic interaction between CQD and (111) planes of Cu_2_O [71]. Ding et al. (2015) reported a superior NEGH sensing electrode with excellent conductivity using Cu_2_O microspheres (MSs) decorated on reduced graphene oxide (RGO). Cu_2_O MSs of different sizes and uniform shapes were obtained on the surface of RGO by varying the mass ratio (1:20 to 1:80) using sodium ascorbate in the presence of sodium hydroxide. The RGO sheets cover the Cu_2_O and act as additional surfactant. This reduces the microsphere size, prevents particle aggregation, protects Cu_2_O MSs and improves the electrochemical stability. The typical reaction method controls the Cu_2_O nanocrystal morphology with the addition of a capping agent and the Cu_2_O MS grows on RGO sheets by the Ostwald ripening mechanism. When the mass ratio was 1:80, the sensor produced the best performance, i.e., a 0.005 to 2.775 mM linear detection range and a 0.0108 mM detection limit for H_2_O_2_ and a 0.001 to 0.419 mM linear detection range and a 7.288 × 10^−4^ M detection limit for glucose. In addition, this sensor showed improved stability with excellent selectivity and good reproducibility because of the extraordinary high surface property of RGO, which reduced the size of Cu_2_O MSs to improve the catalytic activity and the synergetic interaction between RGO and Cu_2_O MSs [72]. In another report, Lu et al. (2016) developed a self-supporting NEGH sensing electrode by modifying 3D copper foam into a pod-like Cu_2_O nanowire array as shown in Figure 1d. The Cu foam acted as a current collector and facilitated charge and mass transfer, while the open framework of the foam provided large amounts of anchoring sites for the deposition of Cu_2_O NWs. The Cu_2_O PLNWs/Cu foam, respectively, showed the sensitivity of 6.6807 mA mM^−1^ cm^−2^ and 1.4773 mA mM^−1^ cm^−2^ to glucose and H_2_O_2_ and detection limits of 0.67 and 1.05 μM. It further exhibited high stability (retained 98.9% of initial response after a week) and resistance to interference studies. The relative standard deviation was 4.61% for six tests for 0.1 mM glucose, substantiating good reproducibility [38] and thus promising that enzymeless glucose and H_2_O_2_ sensors can be developed by manipulating the structural integrity of the Cu-based nanocomposites.

### 3.3. Cobalt Oxide (Co_3_O_4_) and Nickel Oxide (NiO) Nanocomposite

Cobalt oxide exists in three polymorph forms as cobaltous oxide (CoO), cobaltic oxide (Co_2_O_3_), and cobalt oxide (Co_3_O_4_). Among them, Co_3_O_4_ has been studied for non-enzymatic glucose and H_2_O_2_ sensing because of its biocompatibility, and pseudo electro catalytic property. A few research articles are available for the dual sensing of NEGH based on Co_3_O_4_ nanomaterial. The synthesis of Co_3_O_4_ NPs using metal-organic frameworks (MOFs) as a template was investigated by Hou et al. (2012). For this, the Co_3_O_4_ NPs of 20 nm diameter were drop casted on GCE and tested NEGH sensing in alkaline media. The modified electrode also showed efficient practicable performance in human serum for glucose and in disinfectant solution for H_2_O_2_. Overall, the Co_3_O_4_ NPs showed a satisfactory performance when compared with traditional results [73]. Furthermore, Karuppiah et al. (2014) adopted the hydrothermal method to fabricate graphene/Co_3_O_4_-NP composite for the electrochemical sensing of glucose and H_2_O_2_. SEM images revealed a uniform distribution of Co_3_O_4_ nanoparticles on graphene nanoflakes due to the strong interaction of Co-O-C bonds as a result of the highly reactive sp^2^ carbon atoms of the graphene flakes and the electron-rich oxygen species of Co_3_O_4_ nanoparticles. The modified electrode exhibited excellent stability, repeatability and reproducibility [74]. The nickel oxide (NiO) nanomaterial also holds great promise as an electrode material for non-enzymatic sensing due to its low toxicity, excellent electro catalytic activity, and stability. Ni forms hydroxide species (NiOOH) in alkaline medium and catalyzes the analytes’ oxidation during the sensing process. When the Ni-based materials as nanoparticles or nanocomposites are grown on specific substrates, the synergistic effect of particle–substrate combination enhances the efficiency of electro catalytic sensing [75]. Many different ways are adopted to develop the Ni-based sensors, a few of which are discussed in this section. GoO et al. (2014) proposed a conventional electrodeposition technique for NEGH sensing based on Ni (OH)_2_/electro reduced graphene oxide (ERGO)-MWCNTs. In this nanocomposite, graphene oxide (GO) nano sheets served as a surfactant to stabilize MWCNTs, whereas MWNTs functioned as connecting bridges between ERGO sheets and GCE to enhance the electron transfer mechanism, and Ni (OH)_2_ acts as a suitable electro catalyst for glucose and H_2_O_2_ sensing. This sensor exhibited a very high sensitivity due to the synergistic interaction and further confirmed the practical application in urine and milk samples. The glucose showed 106% recovery with a relative standard deviation of 3.72% in urine and, the H_2_O_2_ sensor retained 104.9% with a standard deviation of 2.39% in milk. This work opens new avenues for NEGH sensors as non-enzymatic biosensors [76].

The current review addressed the unique metal oxides, such as Cu_2_O, CuO and NiO Co_3_O_4_, in NEGH sensing. CuO- and Cu_2_O-based nanomaterials are the most popular modified electrodes in NEGH sensing due to its efficient catalytic property, stability and runnable working potential to avoid interference during sensing. However, these electrodes have a few limitations, such as poor conductivity and structural instability during operation. To overcome these limitations and to realize the practical applications, researchers have focused on designing unique morphologies and combined them with carbon/polymer materials. The metal oxide nanocomposite in neutral/acid/alkaline media have performed with high sensitivity and selectivity. In a few reports, different morphologies of CuO electrode have demonstrated high sensitivity, especially with low interference phenomena due to its tunable working potential. Therefore, researchers need to focus on novel metal oxide electrode materials, such as MnO, CeO_2_, TiO_2_ and Fe_3_O_4_/Fe_2_O_3_, for dual sensing of glucose and H_2_O_2_. 

## 4. Metal-Metal Nanocomposites for NEGH Sensing

Bimetallic nanoparticles (BNPs) have been extensively investigated in various applications because of their unique properties, and they are more efficient catalysts than most mono metal NPs. The presence of synergistic interactions between two metals within a bimetallic system can potentially improve NEGH sensing performance and reduce surface poisoning, interfering effects of electrodes and bimetallic structures, including alloys such as Pt-Co, Au-Ag and Pd-Ni. BNP-based sensors have shown better sensing parameters due to enhanced electron transfer and surface area dependent tunable electro catalytic activity. In recent years, BNP-based sensor reports have increased, reflecting a change in the trend of engineered nanomaterials. Bimetallic systems have been combined with graphene/CNT in NEGH sensing, leading to significant advances in this area. 

### 4.1. Platinum Bimetallic Nanocomposite 

Bimetallic platinum-gold nanoparticles are one of the preferable alloys in catalytic and bio-sensing studies. The three-dimensional nano spongy architecture for the PtAu alloy was developed with a size of 5 nm by Wang et al. (2014) and utilised for electrochemical sensing. The nanoporous metals possessed an interconnected network backbone and hollow channels, large internal surface and high electrocatalytic activity. PtAu NP catalysts were obtained at much higher current densities than commercial Pt/C and Pt NPs due to the synergetic catalytic effect of Pt and Au. The homogeneity of PtAu NPs facilitated mass transport and electrical conductivity, leading to enhanced chloride ion resistance, showing high sensitivity, a good detection limit and a wider linear range to H_2_O_2_. However, with glucose, the values were slightly less (0.5 µM detection limit and 0.2–5.4 mM linear range), but comparable stability, durability and selectivity [77]. Though the bi-continuous nano scaled skeletons and interconnected hollow channels within the particle promoted the electrochemical sensing responses, an additional nafion coating, when applied, decreased the selectivity by allowing the interferents to react. 

In order to decrease the cost of the electrode and explore the catalytic activity of Co, Au was replaced with Co and fabricated Nano porous PtCo NPs with a size distribution of 3 nm by dealloying PtCoAl in a mildly alkaline solution. The simple dealloying process pre-defines the nanoparticles’ bimetallic composition without losing the target metal, compared with the traditional chemical synthesis through which the reduction in individual metal salts occurs. The current density for H_2_O_2_ oxidation by the PtCo nanoparticle (0.90–1.2 V) was 10 times higher than that of the Pt/C catalyst due to the catalysis effect on the H_2_O_2_ electro oxidation. Higher scan rates also increased the oxidation current, attributed to the diffusion-controlled process happening on the PtCO alloy. The PtCo alloy showed a high sensitivity response, and wide linear range due to its synergetic electro catalytic activity on electrochemical reactions. Besides, the PtCo alloy also exhibited good anti-interference towards AA, UA, and DA. The authors also reported effective detection of ethanol in addition to glucose and H_2_O_2_ and claimed advantages such as easy preparation, improved precious metal utilization, and large-scale preparation [78]. Furthermore, in the NEGH sensing of Pt BNP, Mei et al. (2016) designed a novel PtNi/MWCNTs nanocomposite using a chemical reduction method. Ni NPs in the PtNi alloy had a dramatic synergetic effect on the electrochemical activity. In contrast, the CNTs enhanced the electro catalytic activity of the alloy and prevented alloy precipitation or aggregation, resulting in the acceleration of electron transfer and enhanced sensitivity. Extraordinarily, the Pt/Ni/MWNCTs-based sensor exhibited superior electro catalytic activity in neutral solutions towards H_2_O_2_ and glucose at a positive working potential of 0.45 V and +0.1 V [79]. To further boost the catalytic and electronic properties of Pt BNP, Mei et al. (2016) have developed a core-shell nanoparticle’s morphology using Pt as a shell and Fe as a core part with carbon (Fe@Pt/C) for the sensing of glucose in human serum samples and H_2_O_2_ in lake water and antibacterial lotion (3%). The Fe@Pt/C core-shell nanoparticles were prepared by spontaneous replacement reactions using Vulcan XC-72 carbon as supportive material. The superb electrical conductivity and great electro catalytic activity of Fe@Pt/C make them sensitive and rapid electrochemical sensing platforms for the reduction of H_2_O_2_ and oxidation of glucose. In fact, the lower electronegativity of Fe compared to Pt changes the electronic properties of Pt and its d-band density is lowered in energy in the Fe@Pt-skin structure. This induces changes in chemisorption energies and increases the number of analyte adsorption active sites. These surface structural and electronic effects (in other words strain and ligand effect) are responsible for the electrochemical sensing mechanisms in response to various analytes. The H_2_O_2_ sensor retained 92% of the current response in 30 days, indicating long-term stability and reproducibility with a relative standard deviation of 1.2%. Practical applicability was also studied by testing the presence of H_2_O_2_ in lake water and antibacterial lotion and glucose in human blood, and in all cases, very similar values were obtained with recorded data. In addition, this sensor also exhibited good reproducibility, long-term stability, and selectivity in the presence of interference compounds [80].

### 4.2. Palladium Bimetallic Nanocomposite 

**Figure 4:**
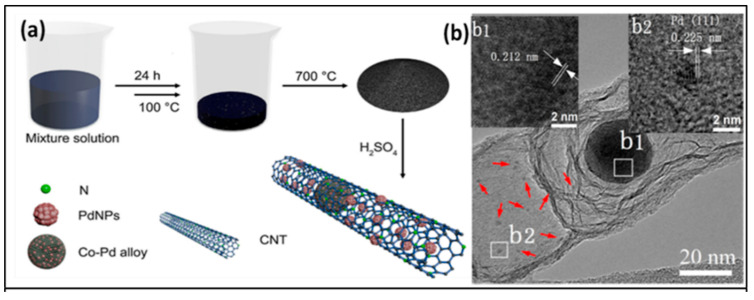
(**a**) Schematic diagram for preparation of Pd-CoCNT; (**b**) HRTEM images of Pd-CoCNT [85].

Three-dimensional and bicontinuous nanospongy PdCr alloy of ligament size, ∼5 nm was reported for the significant effect of Cr in improving the stability and exhibiting synergetic catalytic effect on electro catalytic reactions for NEGH sensing. The as-synthesized PdCr alloy exhibited a wide linear range (0.1 to 1.9 mM) with low detection limit (3.1 μM) towards H_2_O_2_ sensing with no loss in electro catalytic activity after long-term storage for two weeks. The sensor also showed high sensing properties for glucose with wide linear ranges (1–38 mM) [81]. With the same synthesis method, the same research group also developed nano porous PdFe and studied the effect of Fe on the sensing performance of both glucose and H_2_O_2_. Dealloying PdFeAl here also produced similar nanospongy architecture with 5 nm ligament size. When Fe is combined with Pd, the electrochemical properties modify due to the smaller electronegativity of the Fe as opposed to the Pd and enhances the d-band electron density in Pd for the generation of OH_ads_ species on a PdFe nanocomposite surface. The desorption of OH_ads_ or reduction of Pd/Fe generates the active metallic surface for the electro-oxidation of glucose. The added advantage of this sensor is the high resistance to interference species such as Cl^-^ ions, AA, UA, and DA [82]. Using the same dealloying method, Dianyun et al. (2015) generated nanoporous PdNi alloy composite for NEGH sensing. The nanoporous network with hallow interconnections made a bicontinuous skeleton nature for the nanocomposites. The electrochemical parameters revealed a high catalytic activity of as-synthesized PdNi alloy rather than Pd NPs and Pd/C catalyst. This work provided a simple and green route to construct efficient electrodes for glucose and H_2_O_2_ non-enzymatic sensing [83]. Furthermore, researchers combined unique 2D molybdenum disulfide (MoS_2_) nano sheets and the high electro catalytic activity of Au-Pd BNP using a facile thermal co-reduction method to achieve a wide linear range, low detection limit, and good stability. A low working potential of −0.3 V for the reduction of H_2_O_2_ in a neutral solution and the −0.1 V for glucose in alkaline medium was reported using Au-Pd/MoS_2_ nanocomposites. Both glass electrode and MoS_2_ nanosheets did not show any oxidation peak in the presence of glucose, indicating their non-electrocatalytic activity. Strong peak currents of glucose electrooxidation was observed for Au-Pd/MoS_2_ electrode. Glucose on exposure to the electrode surface, is adsorbed on to it due to the dehydrogenation of the anomeric C1 atom. Such adsorbed moieties occupy the Pd active sites and inhibit further electrooxidation of glucose. During a positive potential scan, the Pd-OH species developed in the presence of aqueous NaOH catalyze the adsorbed intermediate oxidation, which makes the Pd active sites free for the direct electrooxidation of glucose. Further positive potential scan decreases the peak current as Pd oxide is formed to inhibit the electrooxidation. The reduction of this Pd oxide occurs during the negative potential scan and almost simultaneously, the surface Pd active sites become available for the electrooxidation process. All these significant reactions are attributed to the synergetic interaction between the MoS_2_ and Au-Pd bimetallic combination [84]. A highly electroactive material was fabricated for the first time without a pretreatment approach based on in situ Pd-Co alloy supported over carbon nanotubes (Pd-CoCNTs) via a one-pot pyrolysis process as shown in Figure 4a. The nanostructure prevented agglomeration due to in situ formation and has much more stability than the previously reported Pd nanocomposite, and the morphology of the TEM image is depicted in Figure 4b. The low concentration of Pd and small size (diameters of 2–4 nm) on Pd-CoCNTs reduced the competition among active sites and resulted in good selectivity, good stability and sensitivity. While the lower concentration and detection limit for glucose were 10 μM and 1 μM, respectively, the sensor exhibited a 0.3 μM detection limit for H_2_O_2_ [85]. 

### 4.3. Copper Bimetallic Nanocomposite 

Noh et al. (2012) fabricated a hierarchical Cu–Co alloy dendrite by electrochemical synthesis. The Co^2+^ ions that formed on the alloy dendrite contributed to glucose oxidation, and Co^3+^ was the main species involved in the reduction of H_2_O_2_ with Cu^2+^ ions contributing to the electrocatalytic process. The major oxidation product/number of an electron that participated in the conversion of glucose was identified to 97% of formate (12-electron oxidation product) and the remaining 3% with other minor products through coulometry and High pressure liquid chromatography–mass spectrometry (HPLC-MS) analysis. The electrochemical properties analyzed at different pH conditions and temperatures achieved a dynamic detection limit, and long-term stability. Compared to the single metal dendrite, the Co bimetallic dendrite enhanced the catalytic property by 10 times [86]. Silver nanodendrites on Cu rods were synthesized by a facile displacement reaction with the absence of any surfactants. In this work, the dendritic Ag structures offered a large surface area for good conductivity of Cu-Ag BNP and reported an ultra-low detection limit for glucose and H_2_O_2_ sensing. The advantage of this work lies in its good reproducibility as the electrode can be regenerated under hydrodynamic conditions without any extra treatment method. Five successive cycles of sensing experiments demonstrated a relative standard deviation of 3.59% to 4.22%. In addition, good selectivity and long-term stability over 30 days were also achieved by the dendrite sensor [87]. Mei et al. (2016) prepared three-dimensional nanoporous copper (Al_75_Cu_25_) and carbon black by dealloying Al-Cu ribbons to make a clean surface highly conductive material. In addition to the low cost and simple preparation, the dealloying process possesses control over structural uniformity of the synthesized materials. Other advantages of the prepared sensor include environmental protection by dealloying and good selectivity through synergistic interaction between the nanoporous copper and carbon black. The sensor operated at a working voltage of 0.6 and 0.75 V (vs. saturated calomel electrode (SCE)) for glucose and H_2_O_2_, respectively. The numerous conducting channels present in carbon black help to transfer electrons and the Cu-carbon black structure allows for electronic transfer between their active sites. The working electrode has a wide linear analytical range, good selectivity, stability, and sensitivity in the positive potential window. The detection limits for glucose and H_2_O_2_ were 2.6 μM and 1.2 μM, respectively. Real-time analysis was also performed in some commercial beverages for glucose and different contact lens solution for H_2_O_2_ and achieved good correlation with existing values [88]. The different combination of bimetallic AuCu, PtCu, and Fe, Ni-CNTs were successfully tested for NEGHS and achieved high sensing parameters [89,90,91]. 

### 4.4. Other Bimetallic Nanocomposite 

Metal nitrides are reported for their applicability in designing glucose and H_2_O_2_ sensors due to their superior electrical conductivity, exceptional redox properties and mechanical strength. Zhou et al. (2017) fabricated a Fe_3_N-Co_2_N nanowire array on carbon cloth, which is an attractive bifunctional catalyst for NEGH sensing because of a large surface area and easy accessibility. This transition metal nitride had metalloid characteristics with superior electrical conductivity and had not yet been reported for NEGH sensing. The fabricated Fe_3_N-Co_2_N/carbon cloth sensor obtained a respective response time of 8 and 2 s for glucose and H_2_O_2_. The prepared sensor exhibited a high selectivity, specificity, and reproducibility [92]. Furthermore, Deepalakshmi et al. (2018) prepared core-shell nanostructures based on nitrogen-doped graphene encapsulated nickel-cobalt nitride (Ni_x_Co_3-x_N/NG) via a simple, scalable, and cost-effective pyrolysis technique. This work suggested that Ni was the best choice in combination with Co as opposed to Fe transition metals for sensing applications. It successfully controlled the molar ratio of Ni/Co to achieve ahigh electrocatalytic activity, and nitrogen-doped graphene provided a high conductive nature and long-term cycling stability of a working electrode, as shown in Figure 1e. Due to the synergistic effect of the NiCo_2_N core and the NG shell, highly sensitive and selective properties were obtained for the electrodes. The practical feasibility of the prepared electrode was tested in human serum, and proved to be efficient for the determination of glucose and H_2_O_2_ [39]. 

Palladium (Pd) nanocomposites have attracted researchers’ interest due to their high electro catalytic activity, lower price, and abundant yield when compared with Au, Ag and Pt materials. Palladium bimetallic nanocomposite improved the electro catalytic performance by modifying the structure, correction in ligand and strain effect. However, the surface of Pd metal can easily be poisoned by chloride and intermediate species and remains unstable during electrochemical reactions leading to a decrease of sensing parameters. Many researchers synthesized Pd alloy in the form of interconnected porous nanostructure using advanced fabrication techniques. The porous channels prepared by the dealloying method are preferred for rich surface chemistry, unique catalytic activity, easy mass and electron transport, unlimited electron conductivity, and synergetic effect. The nano porous Pd-based alloys prepared by the dealloying method, which gained a valid fabrication route to construct highly effective electrochemical sensors and had advantages such as easy handling, no particles aggregation, clean metal surface, and eco-friendliness compared to other reported synthesis methods. Platinum nanoparticles are widely applied in the analysis of non-enzymatic glucose and H_2_O_2_, but there are several limitations like slow kinetics, low sensitivity, and poor selectivity. In a few reports modified platinum with Au, Co, Fe, and Ni and altered surface catalytic activity have been mentioned. Furthermore, these alloys are decorated with carbon/MWCNTs to enhance stability and sensitivity. In the same way, palladium incorporated with transition metals such as Fe, Cr, Ni, Co and noble metal Au has been reported. By considering the effective combination such as CNTs, MOS_2_ etc., specific preparation methods have been applied to overcome the limitations of Pd BNP for sensing both glucose and H_2_O_2_. The bifunctional properties of Cu bimetallic nanocomposites also contributed to the enhancement of NEGH sensing application. The obtained morphological changes offered the best electro catalyst for NEGH sensing. Modifying the morphology of copper-based nanomaterials into nanowires, nanoplates, nanospheres, and nanofibers altered the potential window to avoid etching and interference of electrodes in alkaline solutions and showed significant performance in achieving high electrocatalytic activity and selectivity. Among the various nano morphologies, metallic dendrites structures are attracted in NEGHS due to their high surface area and a high degree of connectivity with the main stem and many side branches. Researchers have further focused on enhancing the surface area of Cu-based bimetallic nanostructures for sensing both glucose and H_2_O_2_. Finally, bimetallic nitrides (BMN) have attracted attention due to their exceptional redox property, superior conductivity, interstitial alloy behavior and exceptional mechanical strength. Among BMN, Co nitrides showed superior electrical conductivity, high chemical stability and extraordinary corrosion resistance. The exchange of nitrogen with oxygen in BMN prefers large electron donating ability for higher electrical conductivity. The BMN in electrochemical reactions suffers from poor stability due to easy oxidation. To overcome these issues, researchers have combined BMN with supporting materials such as graphene, activated carbon and CNTS. Table 3 shows bimetallic nanocomposites in NEGH sensing with their electrochemical performances. 

## 5. Metal/Metal Oxide-Metal Oxide Nanocomposites for NEGHS

A combination of two metal/metal oxides is another effective approach to improve the electro catalytic activity for NEGH sensing [93]. In recent years, researchers have focused on perovskite oxide (ABO_3_)-type nanomaterials as an attractive non-noble metal alternative in the electrochemical field, mainly due to the presence of oxygen vacancies in the crystal structure. Some perovskites have a strong electrocatalytic activity to oxygen reduction and oxidation phenomena and are most suitable for NEGH sensing. Liotta et al. (2015) investigated low cost, commercial carbon screen-printed electrodes (CPEs) and modified perovskite nanomaterials for NEGHS. La_0.6_Sr_0.4_Fe_0.8_O_3-d_ (LSF) and La_0.6_Sr_0.4_Co_0.2_Fe_0.8_O_3-d_ (LSCF) perovskites were synthesized by the citrate method (citric acid/metal ratio = 1:5) in the presence of NH_4_OH at pH 9 to 10. The fabricated modified electrodes showed enhanced oxidation current attributed to the mixed vacancy states of Co and Fe ions, which are accountable for charge transfer in the electro-oxidation of glucose and H_2_O_2_. In addition, the LSCF sensor attained good selectivity due to lower anodic potential in human serum samples [94]. Similarly, Zhang et al. (2012) synthesized LaNi_0.6_Co_0.4_O_3_ (LNC) via a sol-gel method and demonstrated the NEGHS based on LNC/CPEs modified electrode, which avoids the stability problems, complex fabrication process and limited lifetime. LNC NPs showed excellent electrocatalysis to the oxidation of H_2_O_2_ and glucose due to the increased electroactive surface area, intrinsic peroxidase-like activity, and the existence of abundant active sites. The sensor demonstrated good sensitivity and a low level of detection (for H_2_O_2_, the concentration range was 10 nM–100 μM with 1.0 nM detection limit; for glucose, the concentration range was 0.05–200 μM with 8.0 nM detection limit). Moreover, this prepared sensor was able to detect glucose in serum and H_2_O_2_ in toothpaste samples [95]. 

The same research group also prepared novel Co_0.4_Fe_0.6_LaO_3_ (CFL) NPs via a sol-gel method, and the CFL NPs revealed smooth surfaces with uniform thickness and a particle size of 30 to 70 nm. The enhanced electrocatalytic activity of the composite was attributed to the active sites, which are the transition metal ions with partially occupied d orbitals. The Co_0.4_Fe_0.6_LaO_3_ catalyst has transition metals in mixed oxidation states as Fe(II)Co(II)/Fe(III)Co(III), and cyclic electron transfer happens while detecting H_2_O_2_. During the sensing process, the strong oxidizing agents, Fe (III) and Co (III) electrochemically oxidize CFLs to FeOOH and CoOOH. The OH^-^ ions formed along with the species act as the reactive units for glucose sensing. This sensor also offers a fast response, high stability, good reproducibility, and reasonable selectivity. The authors proposed the use of these perovskite structure oxide-based, low-cost, non-enzymatic sensors for public health and environmental applications [96]. In order to increase the performance of perovskite, the rGO has been used for NEGHS application by He et al. (2017). This group synthesized LSC, LSCF, and LNC perovskite via a sol gel process using Ethylenediaminetetraacetic acid (EDTA) citrate as complexing agent. Among them, LSC showed superior electro-oxidation of glucose and H_2_O_2._ This work revealed the possible electro chemical mechanism and its pathways of redox activity and formation of Co^3+^/Co^4+^ redox couple via oxygen vacancies and made a route to elucidate the theoretical framework to design new perovskite sensing electrodes. They finally proposed the combination of perovskite with rGO acting as a unique sensing electrode with notable sensitivity, selectivity, stability, and reproducibility through a synergistic effect [97]. Furthermore, the morphology of perovskite was improved by Wang et al. (2013) based on LaNiO_3_ nanofibers (LNFs) by electro spinning and subsequent calcination and achieved a high surface area because of the porous structure. This type of effective combination of synthesis method and electrochemical sensing developed reliable NEGH biosensors, with an ultra-low detection limit and wide linear range (33.9 nM and 0.05 to 1000 μM for glucose). This LNFs/CPE performed enhanced catalytic activity and high selectivity and sensitivity in the presence of AA, UA, and DA, in addition to the long-term stability [98]. Xia and coworkers (2018) utilized the same electro spinning process without using a template as an added advantage for NEGH sensing application to avoid contamination problems. This group prepared highly porous CuFe_2_O_4_ nanotubes on nickel foam and achieved a high sensitivity and low detection methods in alkaline solutions. The excellent porosity, flexibility, surface area, inner and outer surfaces and good conductivity of the nanotubes provide many active sites and transmission of electrons. This leads to the high electrochemical activity of the material at a typical voltage of 0.5 mV in alkaline media [99]. Furthermore, Ensafi et al. (2016) formulated the NEGH sensor based on Ag/SiO_2_ nanostructures. These were prepared by decorating the surface of organic functionalized SiO_2_ with silver nanoparticles. Modifications of CPE allowed for easier electron transfer when compared to the unmodified electrode as the functionalized SiO_2_/decorated Ag nanoparticles achieved electrocatalytic effects. Synthesized compounds were used to analyze H_2_O_2_ and glucose levels in commercial UHT dairy products and plasma samples, showing a remarkable selectivity towards H_2_O_2_ and glucose levels, respectively [100]. Zhao et al. (2017) exploited a facile hydrothermal technique for the in situ deposition of CuO/rGO on copper foil. Structural and morphological characterization confirmed that the nanocomposite contained three types of interfaces, namely CuO/rGO, rGO/Cu_2_O, and Cu_2_O/Cu. This facilitated redox reactions between GO and the copper foil, resulted in the electrostatic attraction of (+) vely charged copper ions and (–) vely charged rGO. The modified foil achieved an amperometric response of glucose (at 0.45 V vs. SCE) with a low detection limit of 0.10 mM, a linear range of 0.5 to 8.3 mM, an ultrahigh sensitivity of 3401 mA mM^−1^ cm^−2^, and a response time of <0.5 s. With regards to H_2_O_2_ sensing at an applied potential of −0.2V, the modified electrode had a low detection limit of 0.05 mM, a linear range of 0.5 to 9.7 mM, a sensitivity of 366.2 mA mM^−1^ cm^−2^, and a response time of 0.8s. Moreover, the prepared CuO/rGO/Cu_2_O/Cu electrode was applied to detect glucose levels in human serum determined to be 4.86 mM, consistent with the laboratory-based value of 5.01 mM [101]. 

A recent report investigated a one-step anodization process to construct a self-supporting Co_3_O_4_/nanoporous gold (NPG) composite. This electrode effectively worked in alkaline solutions with an ultra-sensitivity of 4470.4 mA mM^−1^ cm^−2^, a low detection limit of 0.085 mM, and a linear range of 2 µM to 2.11 mM for the detection of glucose. With the same electrode, H_2_O_2_ showed a sensitivity of 1338.7 mA mM^−1^ cm^−2^ with a linear range of 20 to 19.1 mM, and both the sensing results are comparable to the hospital laboratory results [102]. Direct growth of nanostructures on Cu foil via a hydrothermal method was the subject of research for generating tubular hierarchical structures. The morphological studies of synthesized Ni (OH)_2_/rGO/Cu_2_O revealed that Cu substrate modified to Cu_2_O with uniform cubic structure and tubular hierarchical structures of Ni (OH)_2_ are grown on the surface of reduced graphene oxide as shown in Figure 1f. The outstanding electro catalytic activity of this material was ascribed to the synergistic interactions of rGO, Ni (OH)_2_, and Cu_2_O. Ni (OH)_2_ structure had promoted the effective diffusion of glucose molecules, while the wrinkled graphene functioned as an excellent electric conductor. The sensor showed remarkable reproducibility and superior stability for long-term applications [40]. Long et al. (2018) reported CuO/NiO hallow nanocomposite via the solvothermal process and subsequent thermal treatment. This work developed core-shell, yolk-shell, or hollow structure of CuO/NiO by adjusting the amount of NiCl_2_ during synthesis. A porous hallow structure showed outstanding electrochemical properties due to the synergetic interaction of CuO and NiO, porous hallow, and large void spaces. The electrode exhibited a high sensitivity towards glucose and for H_2_O_2_. Furthermore, it was applied in human serum to estimate practical feasibility [103]. Wang et al. (2018) produced a novel combination of nano hybrids through a two-step process for NEGH sensing. A particle size of 7 nm ZnO/CoO nanoparticles was decorated over a graphene sheet to achieve high conductivity and abundant active sites. This modified ZnO/CoO/rGO/GCE showed a remarkable selectivity in the presence of AA, UA and KCl, due to the low working potential of the electrode [104]. Lu et al. (2019) fabricated a highly conductive and large surface area electrode for NEGH sensing based on 3D nitrogen-doped graphene hydrogel (NHGH) decorated with NiCo_2_O_4_ nanoflowers using the hydrothermal method. The novel NHGH/NiCo_2_O_4_ nanocomposites performed an excellent electro catalytic activity to detect glucose and H_2_O_2_ due to the presence of abundant active sites. The redox reactions of Co and Ni species in alkaline solution explains the same as mentioned in previously reported work. The sensitivity and high selectivity parameters have been used to detect glucose in blood. Taken together, the results suggest that the hybrid nanocomposite is a promising non-enzymatic electrochemical sensor [105].

From the above discussions, it is clear that researchers have focused on synthesizing bifunctional catalysts using metal (oxide)/metal oxide nanocomposites for NEGH sensing. The morphology, dimensions, surface area, grain and pore size have primarily effected electrochemical NEGH sensing. The peculiar nanostructures such as porous nanotubes, and nano fibers enhance inner and outer surface area, high porosity, excellent flexibility, facilitate the sluggish kinetic process (oxidation of inactive glucose), improve mass and electron transfer between electrode and electrolyte, and maximize the number of active sites. These properties have potentially improved the linear ranges, sensitivity and selectivity. The use of screen-printed electrodes have motivated researchers to elaborate NEGH sensing research in real-time applications and overcome the limitations of portability and instability. A few research groups focused on perovskite-type ferrites for sensing both glucose and H_2_O_2_ because of their fascinating physical and chemical properties such as dual catalytic property (catalysis and peroxidase activity), superior electro catalytic activity, low cost, biocompatibility, rapidness and sensitivity. By considering rGO incorporation, it has solved the aforementioned problems of agglomeration, stability and poor conductivity. The mixed metal oxide exhibited higher conductivity as the activation energy required to transfer electrons from cations is relatively low, which further enhanced the sensing parameters. A significantly smaller number of publications are reported for metal/metal oxide combinations due to wide band gap and homogeneity issues. Based on the literatures, a limited number of transition mixed metal oxide was used for NEGH sensing even though these are low cost and exhibit good electrochemical behavior. Therefore, nanostructures with a high surface area and enhanced charge transfer electrode would be desirable for NEGH sensing in the future. The NEGH sensing parameter with the same electrode-based metal (oxide)/metal oxide nanocomposite are compared in Table 4.

## 6. Future Perspectives

Limited research development has been made with regard to the fabrication of advanced nanomaterials with bifunctional property for NEGHS. Further improved research and development are necessary to make the commercialization of implantable in vivo and portable in vitro NEGHS devices, which require the improvement of practical, affordable, advanced nanomaterial-based electrocatalysts with multifunctional reactivity. The current research review addresses multiple directions for the achievement of non-enzymatic bifunctional electrode platforms. Electrochemical sensing parameters of advanced nanomaterial with bifunctional electrodes are dependent upon the electrode potential, bandgap, surface defects, synergetic effect, and surface area of the nanocomposites. However, the influence of these issues on NEGH sensing is not addressed in the literature and provides opportunities for the future development of biodevices. Since the multienzymatic properties of nanomaterials have attracted wider research interest, the catalytic (glucose) and peroxidase (H_2_O_2_) activity of nanomaterial should be effectively optimized and promoted for the best performance of NEGH sensors. The essential electrochemical mechanism in NEGH sensing with the same electrode material should be established using theoretical and analytical models with relevant laboratory experiments. Current studies on NEGH sensors mostly focus on the electrocatalyst performance of advanced nanomaterials and limit the understanding of the influence of nanomaterial morphology on glucose and H_2_O_2_ quantification and the interaction with bio-analytes. To overcome this, researchers should focus on the development of nanomaterials in different morphologies, such as dots, tubes, fibers, spheres, and core-shells, and a detailed study should be undertaken to improve the surface area and conductivity, which could have a positive influence on the development of NEGH sensors. The modified electrodes show catalytic activity in acidic or basic conditions, which limit the practical application of NEGH sensors. In this context, studies must be done on the oxidation and reduction mechanisms at neutral pH conditions by considering novel nanomaterials. The use of biopolymers as bio-catalytic centers are tolerable to achieve highly sensitive and selective NEGH sensors, and distinct consideration should be given to building electrode platforms with improved robustness and enhanced electro catalytic activity. NEGH sensor-based nanomaterials as catalysts have been demonstrated to be very reasonable; conversely, it is essential to design new schemes for the synthesis, functionalization, and fabrication of nanomaterials to acquire more accurate quantification of glucose and H_2_O_2_. Several sequential steps involved in the preparation of electrodes for a conventional modified electrode based on NEGH sensing, including cumbersome electrode cleaning, polishing and washing, binder and solvent selection, catalyst preparation, and loading process, have increased the time and cost of NEGH sensing electrodes. Furthermore, to establish contact between the working electrode and catalyst using a binder remains another challenge for the performance of NEGH sensing. This could be avoided by developing binder-free, freestanding bare electrodes, ink/screen printed electrodes and the in situ fabrication/modification of advanced nanomaterials as modified electrodes that make possible the preparation of disposable NEGH sensing electrodes. Moreover, another compelling research direction is in the preparation of metal/metal oxide morphologies with emerging carbon materials (g-C_3_N_4_, graphene, CNTs, black phosphorous, and activated carbon, etc.) to form new functional materials. For commercialization, an important prospect is the prolongation of lifetime of the sensors, even though the non-enzymatic sensors are more stable than enzymatic sensors, they lack in the corrosion property/unstable in humid conditions, which requires researchers to focus on anticorrosive nanomaterials. Current challenges in improving efficiency of the NEGH sensors can be overcome by optimizing the selectivity, working potential, linearity, sensitivity and working pH conditions. Though some NEGH sensors are good in neutral pH conditions with low detection limits, their linear range of detection may be questionable. The low detection range sensors are not useful in day-to-day diabetes management and hence few reports have been applied in various real-time applications such as sensing in antibiotic lotions, milk, and glucose-based fuel cells, etc. The selectivity of NEGH remains a huge problem, which means that the oxidation of interference compounds such as AA, DA, and UA chlorine ions and other carbohydrates at the same working potential affects the glucose and H_2_O_2_ determinations. Transition metal/metal-oxide-based sensors have shown significant progress in selectivity issues and electrode fouling problems due to reasonable isoelectric point values. From the reported literature on NEGH sensors the sensitivity was improved using different strategies and the novel combination of nanomaterials. Sensor sensitivity is dependent on on working potential, electro kinetics and electrolyte conditions. However, different research groups have performed sensing under their own optimized conditions, which necessitates a uniform protocol for sensing operations. In addition, to improving the sensitivity by optimizing the properties of advanced nanomaterials, the selectivity performance should be more focused to achieve stability, repeatability, and practical evaluation of glucose and H_2_O_2_. The dual in-line sensor requires a clear mechanism with suitable working conditions in neutral pH. The use of the same electrode material for multiple applications is essential to reduce the cost and will make commercialization easy. The dual sensor requires a clear electro catalytic mechanism for sustainable development, and it can be achieved by operating the electrodes at the same working potential (positive/negative). In short, the bifunctional, electro-catalyst-based NEGH sensing technology must be extended from the laboratory to the field by proper implementation to boost sustainable electronic devices.

## 7. Conclusions

Non-enzymatic glucose and H_2_O_2_ (NEGH) electrochemical sensors can be developed based on metals, metal oxides, bimetallic/metal oxide insole, and in combinations with graphene, graphene oxide, carbon nanotubes, graphitic carbon nitride, and polyaniline materials. Several important parameters, such as working potentials, sensitivity, linear range, and selectivity need to be considered for the development of better NEGH sensing, and advanced nanomaterials have been recently suggested as an effective electrocatalyst. This review provides a vital summary of previous NEGH sensing studies and discusses the current state and comparative characteristics of different NEGH sensing modified electrodes to detect both glucose and H_2_O_2_ in dual in-line monitoring systems. The metal nanocomposites exhibited excellent catalytic activity and notable NEGH sensing performances in terms of detection limits and linear ranges of glucose and H_2_O_2_. Especially, the development of transition metal chalcogenides (TMDs) such as NiN_2_, CoN_2_, Cu_2_S, CoS, and V_2_S has overcome the limitations of poor conductivity, less active sites, low stability, low electron transfer, wide band gap and over potential issues. The metal oxide nanocomposites are low cost and highly tolerable in neutral pH conditions compared to metal nanocomposites. However, the metal oxides, such as Cu_2_O, CuO, NiO and Co_3_O_4_, in NEGH sensing have limitations like poor conductivity and structural instability during operation. These limitations could be overcome by designing unique morphologies, which show excellent performance with high sensitivity and selectivity. Bimetallic nanocomposites generally have better electro-catalytic activity and conductivity compared to other nanocomposites. Bimetallic nanocomposites with a porous nanostructure are mostly fabricated by using the dealloying method for NEGH sensing. Modifying the morphology of bimetallic into nanowires/plates/spheres, and nanofibers altered the potential window to avoid interference of electrodes in achieving high selectivity. Among bimetallic nanocomposites, bimetallic nitrides (BMN) have attracted attention due to their exceptional redox property, superior conductivity, and high corrosion resistance and mechanical strength. Screen-printed electrodes modified metal (oxide)/metal oxide nanocomposites and enhanced electrochemical NEGH sensing, has promoted research in real-time applications and overcame the limitations of portability and instability. The perovskite-type ferrites with rGO solved agglomeration, stability and poor conductivity issues and improved mass and electron transfer between electrode and electrolyte to further enhance linear ranges, sensitivity and selectivity. Even though substantial improvements in NEGH sensors have been made based on exploration of carbon and non-carbon-based nanocomposites, additional efforts are essential to deeply understand the mechanism of glucose oxidation and reduction/oxidation of H_2_O_2_ and NEGH sensing at the same working potential, and to further improve the optimization of sensing parameters in real-time applications. This comprehensive review aims to strengthen the understanding of nanomaterials for NEGH sensing and provide a fundamental foundation to explore novel nanomaterials and innovative ideas to revolutionize the sensing of both glucose and H_2_O_2_ leading to commercialization and clinical application of NEGH sensors.

## Figures and Tables

**Table 1:** Performances of non-enzymatic glucose and H_2_O_2_ electrochemical sensors based on metal nanocomposites.

Electrode Material	Analyte	Sensitivity (uA·mM^−1^·cm^−2^)	Linear Range (μM—mM)	Detection Limit (μM)	Working Potential (V)	Stability (30 Days)	Repeatability (RSD)	Reproducibility (RSD)	Real-Time Application	Ref
AuNBP/MWCNTs /GCE	Glucose	101.2	10 to 36.7	3.0	0.15	98.0%	-	2.3	Human serum	[35]
	H_2_O_2_	170.6	5.0 to 47.3	1.5	−0.50	94.7%	-	3.5	Antibacterial lotion	
Ag–CNx/GCE	Glucose	97	0.001 to 0.1	0.6	0.7	-	-		-	[48]
	H_2_O_2_	1.7 × 10^4^	0.005 to 0.35	0.0045	−0.7	-	-		-	
PCF Cu NP/GCE	Glucose	-	6.6 to 1.3	2.2	0.35	96% (60 days)		3.5	human urine	[49]
	H_2_O_2_	-	8.3 to 1.5	2.7	0	-		3.8	antiseptic solution	
Graphene/pectin-Cu NPs/GCE	Glucose	0.0457	10 to 0.0055	2.1	0.40	6.83%	2.35	2.82	Human serum	[50]
	H_2_O_2_	0.391	1 to 1	0.35	−0.20	5.3%	2.52	2.92	Contact lens solution	
Cu_2_S NRs@Cu foam	Glucose	11 750.8	0.2 to 0.63	0.07	0.45	94.8 (14 days)	3.26	2.12	Human serum	[51]
	H_2_O_2_	745	0.25 to 5	0.12	−0.2	95.6 (14 days)	0.49	2.03	-	
PCF Ni NP/GCE	Glucose	42.3 (uA/mM)	1.6 to 1.4	0.53	0.50	-	1.7	-	-	[52]
	H_2_O_2_	43 (uA/mM)	3.3 to 1.7	1.10	0.50	-	1.8	-	-	
3D flower-like Ni_7_S_6_/GCE	Glucose	271.80	5 to 3.70	0.15	0.45	97.1	-	1.33	Human serum	[53]
	H_2_O_2_	37.77	5 to 20.50	0.15	−0.35	95.8	-	0.92	Antibacterial lotion	
Ni_5.5_Co_1.5_S_6_/GCE	Glucose	494.58	5 to 0.50	0.15	0.425	89.9	-	1.2	Human serum	[54]
	H_2_O_2_	94.79	5 to 15.09	0.15	−0.35	95.2	-	2.1	Antibacterial lotion	
Ni_3_N/GA/GCE	Glucose	905.6	0.1 to 7.64	0.04	0.55	93	-	4.6	Human serum	[36]
	H_2_O_2_	101.9	5 to 75.13	1.80	−0.4	95.2 (15 days)	-	3.3	Human serum	
CoS/GCE	Glucose	28.44	1200 to 10.20	1.5	0.20	-	-	-	Antibacterial lotion	[55]
	H_2_O_2_	17.4	5 to 14.82	1.5	−0.40	-	-	-	Human serum	
3D N-Co-CNT@NG/GCE	Glucose	9.05	2.5 to 10.83	0.1	0.32	94.68	3.3	3.8	Human serum	[37]
	H_2_O_2_	28.66	2.0 to 7.449	2.0	−0.04	96.49 (operational stability)	-	4.2	Human serum	
Co_3_N NW/titanium mess	Glucose	3325.6	0.1 to 2.5	0.05	0.60	91.5	-	4.3	Human serum	[56]
	H_2_O_2_	139.9	1 to 12	0.48	−0.20	92.1	-	5.2	-	
Co/TPEG2/BAPc/MWCNTs/GCE	Glucose	1.970	5 to 0.05 (M/L)	12.5 (M/L)	0.50	-	1.9	2.1	Human serum	[57]
	H_2_O_2_	0.162	5 to 0.05 (M/L)	10 (M/L)	−0.50	-	-	-	Contact lens solutions	
Cu@N-Chit–CNTs	Glucose	-	0.5 to 1	0.05	0.50	93	-	-	Human serum	[58]
	H_2_O_2_	-	0.1 to 1	0.025	−0.25	92	-	-	Processed milk	
Pt/OMCs/Nafion/GC	Glucose	16.69	500 to 4.5	130	−0.08	93.2 (14 days)	-	7.4	-	[59]
	H_2_O_2_	173.4	2 to 4.212	1.2	−0.10	94.6 (14 days)	-	5.2	-	
VO(acac)_2_–PATP–Au/GCE	Glucose	120.24	1 to 0.5	0.1	0.65	100 (20 days)	-	0.2	Human blood	[60]
	H_2_O_2_	326.66	0.02 to 0.9	0.03	−0.11	100 (20 days)	-	0.3	Milk	
VS_2_/GCE	Glucose	41.96	0.5 to 3.0	0.211	-	92% (20 days)	-	2.7	human serum	[61]
	H_2_O_2_	37.96	0.5 to 2.5	0.224	−0.75	-	-	-	Hair dye and Human urine	
g-C_3_N_4_/GCE	Glucose	-	1000 to 12	11	−0.81	81% (40 days)	-	-	-	[62]
	H_2_O_2_	-	100 to 90	2.0	−0.30	-	-	-	-	

**Table 2:** Performance of non-enzymatic glucose and H_2_O_2_ electrochemical sensors based on metal oxide nanocomposites.

Electrode Material	Analytes	Sensitivity (uA·mM^−1^·cm^−2^)	Linear Range (μM–mM)	Detection Limit (μM)	Working Potential (V)	Stability (30 Days)	Repeatability (RSD %)	Reproducibility (RSD %)	Real-Time Application	Ref
CuO nanoflowers/GCE	Glucose	1086.34	1 to 2.79	0.12	0.5	85.40	1.37	4.28	Urine	[63]
	H_2_O_2_	956.69	5 to 14.07	0.85	−0.4	89.77	2.69	5.38	Milk	
CuO-Tyr Modified electrode	Glucose	9.02	900 to 16	20	0.60	97	-	2.5	-	[64]
	H_2_O_2_	2.72	100 to 36	2	−0.25	-	-	-	-	
CuO/PANI/GCE	Glucose	-	1 to 9.899	0.45	0.3	-	-	-	-	[65]
	H_2_O_2_	-	5 to 9.255	0.11	−0.2	-	-	-	-	
CuO nanorods/FTO	Glucose	1319	5 to 0.825	-	0.55	80	-	-	-	[66]
	H_2_O_2_	84.89	250 to 18.75	-	−0.5	70	-	-	-	
Porous CuO/GCE	Glucose	−70.8	1.5 to 0.5	0.8	0.60	88.6 (21 days)	-	-	-	[67]
	H_2_O_2_	50.6	5 to 1.5	1.5	−0.20	87 (21 days)	-	-	-	
Cu_2_O/GCE	Glucose	-	50 to 1.1	47.2	0.50	-	-	-	-	[68]
	H_2_O_2_	3.693	-	0.039	−0.20	-	-	-	-	
MCHNs/GCE	Glucose	52.5	1 to 1.7	0.87	0.6	-	-	-	-	[69]
	H_2_O_2_	156.6	2 to 0.15	1.03	−0.3	-	-	-	-	
Cu_2_O/GNs	Glucose	0.285	300 to 3.3	3.3	0.60	-	-	-	-	[70]
	H_2_O_2_	-	300 to 7.8	20.8	−0.40	-	-	-	-	
CQDs/octahedral Cu_2_O/GCE	Glucose	0.298	20 to 4.3	8.4	0.60	High stability	-	-	Human serum	[71]
	H_2_O_2_	0.13	5 to 5.3	2.8	−0.2	High stability	-	-	-	
Cu_2_OMS–RGO/GCE	Glucose	-	1 to 0.419	0.73	0.6	87.6	-	-	-	[72]
	H_2_O_2_	-	50 to 2.775	10.8	−0.24	89	-	-	-	
Cu_2_O PLNWs/Cu foam	Glucose	6680.7	1 to 1.8	0.67	0.5	98.9 (7 days)	4.61	2.57	-	[38]
	H_2_O_2_	1477.3	5 to 1.77	0.13	−0.3	98.4 (7 days)	0.59	1.28	-	
Co_3_O_4_ NPs/GCE	Glucose	520.7	5 to 0.8	0.13	0.59	-	-	-	Human serum	[73]
	H_2_O_2_	107.4	-	0.81	+0.42	-	-	-	Disinfectant	
GF/Co_3_O4-NPs	Glucose	13.52	500 to 16.5	50.0	−0.55	89 (9 days)	3.9	3.7	-	[74]
	H_2_O_2_	1.14	0.2 to 0.211	0.06	−0.48	97.3 (9 h)	3.2	2.2	-	
ITO/NiO	Glucose	1013.76	2 to 0.29	4.6	0.5	80 (15 days)	-	3	Human serum	[75]
	H_2_O_2_	82.73	10 to 0.87	5.2	−0.46	90 (15 days)	-	3.5	-	
Ni(OH)_2_/ERGO-MWCNTs/GCE	Glucose	2042	1 to 1.5	2.7	0.54		5.9	2.8	Urine	[76]
	H_2_O_2_	711	20 to 9.05	4.0	0.2		6.1	2.8	Milk	

**Table 3:** Performance of non-enzymatic glucose and H_2_O_2_ electrochemical sensors based on metal/metal nanocomposites.

Electrode Material	Analyte	Sensitivity (uA·mM^−1^·cm^−2^)	Linear Range (μM–mM)	Detection Limit (μM)	Working Potential (V)	Stability (30 Days)	Repeatability (RSD %)	Reproducibility (RSD %)	Real-Time Application	Ref
**PtAu NPs/GCE**	Glucose	-	200 to 5.4	0.5	0.6	High (14 days)	2.43	2.97	-	[77]
	H_2_O_2_	-	50 to 2.75	0.1	+0.7	95.9 (13 days)	2.61	3.02	-	
**PtCo NPs/GCE**	Glucose	0.499	50 to 3	0.1	0.6	-	-	-	-	[78]
	H_2_O_2_	-	50 to 0.8	1.0	+0.70	93 (14 days)	2.1	3.4	-	
**Fe@Pt core shell/GCE**	Glucose	11.75	1000 to 16	300	−0.15				Human serum	[79]
	H_2_O_2_	218.97	2.5 to 41.605	0.75	−0.40	92		1.2	Antibacterial lotion and lake water	
**PtNi/MWCNTs/GCE**	Glucose	85,910.0	0.1 to 9.0	0.03	0.1	96.9	-	0.88	Human serum	[80]
	H_2_O_2_	2123.10	0.2 to 24.6	0.06	−0.45	97.9	-	2.2	Lake water	
**PdCr NPs/GCE**	Glucose	0.75	1000 to 38	1.8	0.35	High stability	-	-	-	[81]
	H_2_O_2_	72	100 to 1.9	3.1	1.2	93.1 (14 days)	1.7	3.2	-	
**PdFe NPs/GCE**	Glucose	2.7	1000 to 32	1.6	0.35	High stability	-	-	-	[82]
	H_2_O_2_	38.72	500 to 6.0	2.1	+0.9	95.9 (13 days)	2.3	3.1	-	
**NP-PdNi/GCE**	Glucose	0.75	1000 to 25.00	1.90	0.35	High stability	-	-	-	[83]
	H_2_O_2_	208.60	50 to 1.00	2.10	+1.0	91.5 (operational stability 2000 s	-	3.2	-	
**Au-Pd/MoS_2_/GCE**	Glucose	-	500 to 20	400	−0.1	High (15 days)	4.4	8.2	-	[84]
	H_2_O_2_	184.9	0.8 to 10	0.16	−0.1	98 (15 days)	9.0	7.5	-	
**Pd-CoCNTs/GCE**	Glucose	3.77	10 to 2.4	1	0.5	88.8 (4 days)	-	7.3	Human serum	[85]
	H_2_O_2_	-	2100 to 10.1	0.3	−0.15	-	-	-	-	
**Cu-Co alloy/GCE**	Glucose	-	0.5 to 14	0.1	0.65	95 (3 Months)	-	-	Human serum	[86]
	H_2_O_2_	-	1.0 to 11	0.75	−0.40	95 (3 Months)	-	-	-	
**Ag NDS/CRE/GCE**	Glucose	728.2	0.2 to 7.4	0.1	0.6	High	3.59	4.22	-	[87]
	H_2_O_2_	273.3	0.2 to 19.2	0.1	−0.3	-	-	-	-	
**Nafion/NPC-CB/GCE**	Glucose	33.75	6 to 3.369	2.6	0.6	High (12 days)	12.86	-	Beverage	[88]
	H_2_O_2_	3.914	3 to 2.238	1.2	0.75	-	-	-	Contact lens solution	
**AuCu alloy NPs**	Glucose	339.35	250 to 10	16.62	0.5					[89]
	H_2_O_2_	133.74	50 to 10	10.93	−0.40					
**np-PtCu**	Glucose	-	10 to 2.0	0.1	0.4					[90]
	H_2_O_2_	-	10 to 1.7	0.1	0.7					
**Fe, Ni/CNTs/GCE**	Glucose	-	-	1.23	-	High	-	Good	-	[91]
	H_2_O_2_	-	-	16.89	-	-	-	Good	-	
**Fe_3_N-Co_2_N/CC**	Glucose	4333.7	0.1 to 1	0.077	0.55	88.7	-	4.8	Human serum	[92]
	H_2_O_2_	2273.8	0.15 to 8	0.059	−0.20	90.2	-	3.9	-	
**NixCo_3−x_N/NG/GCE**	Glucose	1803	2.008 to 7.15	0.05	0.45	92.31 (45 days)	-	2.6	Human serum	[39]
	H_2_O_2_	2848.73	0.2 to 3.4985	0.2	0.0	91.05 (45 days)		3.1	Human serum	

**Table 4:** Sensing parameters of non-enzymatic glucose and H_2_O_2_ electrochemical sensors based on metal/metal oxide nanocomposites.

Electrode Material	Analyte	Sensitivity (uA·mM^−1^·cm^−2^)	Linear Range (μM–mM)	Detection Limit (μM)	Working Potential (V)	Stability (30 Days)	Repeatability (RSD %)	Reproducibility (RSD %)	Real-Time Application	Ref
**Ni/NiO@C**	Glucose	1291	10 to 10	0.116	-					[93]
	H_2_O_2_	32.09	Up to 80.7	0.9	-					
**La_0.6_Sr_0.4_Co_0.2_Fe_0.8_O_3-δ_/CPE**	Glucose	285	0 to 0.2	7	0.50	-	-	-	-	[94]
	H_2_O_2_	580	0 to 3	5	0.30	-	-	-	-	
**LaNi_0.6_Co_0.4_O_3/_CPE**	Glucose	643	0.05 to 0.2	0.008	0.55	96.2 (20 days)	-	3.01	Human serum	[95]
	H_2_O_2_	1813	0.01 to 0.1	0.001	+0.55	96.7 (20 days)	-	2.6	Toothpaste	
**Co_0.4_Fe_0.6_LaO_3_/CPE**	Glucose	1013.8	5 to 0.5	0.01	0.55	92.6 (21 days)	-	2.7	-	[96]
	H_2_O_2_	2376.7	0.01 to 0.8	0.002	+0.55	95.1 (21 days)	-	3.16	-	
**LSC+RGO/GCE**	Glucose	330	2 to 3.35	0.063	0.60	-	-	-	-	[97]
	H_2_O_2_	500	0.2 to 3.35	0.05	+0.30	-	-	-	-	
**LNFs/CPE**	Glucose	42.321	1 to 1	0.32	0.60	92.9 (28 days)	-	5.23	-	[98]
	H_2_O_2_	1135.88	0.05 to 1	0.033	+0.60	94.6 (28 days)	-	3.18	-	
**CuFe_2_O_4_ nanotubes/Ni Foam**	Glucose	1239	20 to 5.5	0.22	0.55	102.5 (15 days)	7.4	11	-	[99]
	H_2_O_2_	219.4	500 to 25	0.22	+0.55	115.2	7.4	11	-	
**Ag-SiO_2_/CPE**	Glucose	-	1.43 to 3.202	0.33	0.60	-	-	<5.0	Blood plasma	[100]
	H_2_O_2_	31.9	1.0 to 1.618	0.094	−0.35	-	-	-	Milk	
**CuO/rGO/Cu_2_O/Cu**	Glucose	3401.1	0.5 to 8.266	0.1	0.65	-	-	-	human serum	[101]
	H_2_O_2_	366.22	0.5 to 9.7	0.05	−0.30	-	-	-	-	
**Co_3_O_4_/NPG**	Glucose	4470.4	2 to 2.11	0.085	0.50	87.4 (21 days)	3.9	5.02	human serum	[102]
	H_2_O_2_	230	10 to 1.05	1.4	−0.30	-	4.4	-	-	
**Ni(OH)_2_/RGO/Cu_2_O@Cu**	Glucose	5350	0.5 to 7.67	0.35 μM	0.65	93.8 (14 days)	5.66	-	human serum	[40]
	H_2_O_2_	1706.3	0.5 to 7.5	0.2 μM	0.60	94.5 (14 days)	4.35	-	-	
**CuO_x_/NiO_y_/GCEs**	Glucose	2043	0.20 to 2.5	0.08	0.60	93.6 (14 days)	2.4	3.1	human serum	[103]
	H_2_O_2_	271.1	0.30 to 9.0	0.09	−0.35	-	3.8	5.5	-	
**ZnO−CoO/rGO−GCE**	Glucose	168.7	10 to 11.205	1.3	0.45	94.4 (14 days)	-	4.13	-	[104]
	H_2_O_2_	183.3	25 to 11.1	0.44	−0.20	91.3 (14 days)	-	2.91	-	
**NHGH/NiCo_2_O_4_**	Glucose	2072	5 to 10.95	0.39	0.50	92.5 (28 days)	5.27	8.35	human serum	[105]
	H_2_O_2_	-	1 to 0.51	0.136	+0.50	-	-	-	-	

## Abbreviations

Diabetes mellitus (DM); Non-enzymatic glucose and H_2_O_2_ (NEGH); enzymatic glucose and H_2_O_2_ sensing (EGHS); Multiwalled carbon nanotubes (MWCNTs); Gold Nanobipyramids (AuNBP); Graphitic carbon nitride quantum dot (g-CNQD); Silver nanoparticle (Ag NPs); Sodium hydroxide (NaOH); paraffin wax-impregnated graphite electrode (PIGE); Glassy carbon electrode (GCE); Copper nanoparticle (Cu NPs); Copper sulphide nano rods (Cu_2_S NRs); Nickel nitride (Ni_3_N); Graphene aerogels (GA); Cobalt sulphide (CoS); Three dimensional (3D); Nitrogen (N_2_); Nitrogen doped graphene (NG); cobalt nitrate nanowire (Co_3_N NW); Ammonia (NH_3_); Cobalt(II) nitrate hexahydrate (Co (NO_3_)_2_.6H_2_O); Ammonium fluoride (NH_4_F); Cobalt (Co); Nickel (Ni); Ascorbic acid (AA); Uric acid (UA); L-Dopamine (L-dopa); Sodium, Potassium, Chlorine ions (Na^+^, K^+^ and Cl^-^ ions); vanadium sulfide (VS_2_); graphitic carbon nitrate (g-C_3_N_4_); Gold (Au), Vanadium (V); Vanadium sulphide (V_2_S); Tetra-Cobalt(II) carboxamide-PEG2-biotin phthalocyanine (CoTPEG2BAPc); Ordered mesoporous silica (OMCs); bis(acetylacetonate) oxo vanadium (VO(acac)_2_); 4-(pyridine-40-amido) thiol phenol (PATP); Copper oxide (CuO); Cuprous oxide (Cu_2_O); Tetraamminecopper(II) sulfate monohydrate (Cu(NH_3_)_4_^2+^); Copper hydroxide (Cu(OH)_2_); Polyamic acid (PAA); Polyaniline (PANI); One dimensional (1D); X-ray diffraction (XRD); Ti mesh (TM); poly(acrylic acid) (PAA); High pressure Liquid chromatography–mass spectrometry (HPLC-MS); saturated calomel electrode (SCE); Ethylenediaminetetraacetic acid (EDTA); Fluorine doped Tin Oxide (FTO); Urea (UR); Sucrose (SU); Sodium borohydride (NaBH_4_); polyvinyl pyrrolidone (PVP); N, N-dimethylformamide (DMF); Copper chloride (CuCl_2_); Copper sulfate (CuSO_4_); Potassium hydroxide (KOH); metal-organic framework (MOFs); Electro reduced grapheme oxide (ERGO); Platinum (Pt); Palladium (Pd); Chromium (Cr); Iron (Fe); Aluminium (Al); Molybdenum disulfide (MoS_2_); Iron nitride (Fe_3_N); Carbon screen-printed electrodes (CPEs); La_0.6_Sr_0.4_Fe_0.8_O_3-d_ (LSF); La_0.6_Sr_0.4_Co_0.2_Fe_0.8_O_3-d_ (LSCF); LaNi_0.6_Co_0.4_O_3_ (LNC); Co_0.4_Fe_0.6_LaO_3_ (CFL); LaNiO_3_ Nanofibers (LNFs); Nanoporous gold (NPG); Silicon dioxide (SiO_2_); Zinc oxide (ZnO)
